# Habitat fragmentation and its lasting impact on Earth’s ecosystems

**DOI:** 10.1126/sciadv.1500052

**Published:** 2015-03-20

**Authors:** Nick M. Haddad, Lars A. Brudvig, Jean Clobert, Kendi F. Davies, Andrew Gonzalez, Robert D. Holt, Thomas E. Lovejoy, Joseph O. Sexton, Mike P. Austin, Cathy D. Collins, William M. Cook, Ellen I. Damschen, Robert M. Ewers, Bryan L. Foster, Clinton N. Jenkins, Andrew J. King, William F. Laurance, Douglas J. Levey, Chris R. Margules, Brett A. Melbourne, A. O. Nicholls, John L. Orrock, Dan-Xia Song, John R. Townshend

**Affiliations:** 1 Department of Biological Sciences, North Carolina State University, Raleigh, NC 27695, USA.; 2 Department of Plant Biology, Michigan State University, East Lansing, MI 48824–1312, USA.; 3 Station d’Ecologie Expérimentale du CNRS a Moulis USR 2936, Moulis, 09200 Saint-Girons, France.; 4 Department of Ecology and Evolutionary Biology, UCB 334, University of Colorado, Boulder, CO 80309, USA.; 5 Department of Biology, McGill University, Montreal, Quebec H3A 1B1, Canada.; 6 Department of Biology, University of Florida, Gainesville, FL 32611, USA.; 7 Department of Environmental Science and Policy, George Mason University, Fairfax, VA 22030, USA.; 8 Global Land Cover Facility, Department of Geographical Sciences, University of Maryland, College Park, MD 20702, USA.; 9 CSIRO Land and Water Flagship, GPO Box 1700, Canberra, Australian Capital Territory 2601, Australia.; 10 Department of Biology, Colby College, 5746 Mayflower Hill, Waterville, ME 04901, USA.; 11 Department of Biological Sciences, St. Cloud State University, St. Cloud, MN 56301, USA.; 12 Department of Zoology, University of Wisconsin, Madison, WI 53706, USA.; 13 Department of Life Sciences, Imperial College London, Silwood Park Campus, Buckhurst Road, Ascot, Berkshire SL5 7PY, UK.; 14 Department of Ecology and Evolutionary Biology and Kansas Biological Survey, University of Kansas, 2101 Constant Avenue, Lawrence, KS 66047–3759, USA.; 15 Instituto de Pesquisas Ecológicas, Rod. Dom Pedro I, km 47, Caixa Postal 47, Nazaré Paulista, São Paulo 12960-000, Brazil.; 16 Centre for Tropical Environmental and Sustainability Science and College of Marine and Environmental Sciences, James Cook University, Cairns, Queensland 4878, Australia.; 17 National Science Foundation, Arlington, VA 22230, USA.; 18 Centre for Tropical Environmental and Sustainability Science, School of Earth and Environmental Sciences, James Cook University, Cairns 4878, Australia.; 19 Research Center for Climate Change, University of Indonesia, Kota Depok, Java Barat 16424, Indonesia.; 20 The Institute for Land, Water and Society, Charles Sturt University, Thurgoona Campus, Albury, New South Wales 2640, Australia.

## Abstract

We conducted an analysis of global forest cover to reveal that 70% of remaining forest is within 1 km of the forest’s edge, subject to the degrading effects of fragmentation. A synthesis of fragmentation experiments spanning multiple biomes and scales, five continents, and 35 years demonstrates that habitat fragmentation reduces biodiversity by 13 to 75% and impairs key ecosystem functions by decreasing biomass and altering nutrient cycles. Effects are greatest in the smallest and most isolated fragments, and they magnify with the passage of time. These findings indicate an urgent need for conservation and restoration measures to improve landscape connectivity, which will reduce extinction rates and help maintain ecosystem services.

## INTRODUCTION

Destruction and degradation of natural ecosystems are the primary causes of declines in global biodiversity (*1*, *2*). Habitat destruction typically leads to fragmentation, the division of habitat into smaller and more isolated fragments separated by a matrix of human-transformed land cover. The loss of area, increase in isolation, and greater exposure to human land uses along fragment edges initiate long-term changes to the structure and function of the remaining fragments (*3*).

Ecologists agree that habitat destruction is detrimental to the maintenance of biodiversity, but they disagree—often strongly—on the extent to which fragmentation itself is to blame (*4*, *5*). Early hypotheses based on the biogeography of oceanic islands (*6*) provided a theoretical framework to understand fragmentation’s effect on extinction in terrestrial landscapes composed of “islands” of natural habitat scattered across a “sea” of human-transformed habitat. Central to the controversy has been a lingering uncertainty about the role of decreased fragment size and increased isolation relative to the widespread and pervasive effects of habitat loss in explaining declines in biodiversity and the degradation of ecosystems (*7*). Observational studies of the effects of fragmentation have often magnified the controversy because inference from nonmanipulative studies is limited to correlation and because they have individually often considered only single aspects of fragmentation (for example, edge, isolation, and area) (*8*). However, together with these correlative observations, experimental studies reveal that fragmentation has multiple simultaneous effects that are interwoven in complex ways and that operate over potentially long time scales (*9*).

Here, we draw on findings of the world’s largest and longest-running fragmentation experiments that span 35 years and disparate biomes on five continents. Their rigorous designs and long-term implementation overcome many limitations of observational studies. In particular, by manipulating and isolating individual aspects of fragmentation while controlling for others, and by doing so on entire ecosystems, they provide a powerful way to disentangle cause and effect in fragmented landscapes. Here, we present experimental evidence of unexpected long-term ecological changes caused by habitat fragmentation.

Highlighting one ecosystem type as an example, we first present a global analysis of the fragmentation of forest ecosystems, quantifying for the first time the global hotspots of intensive historical fragmentation. We then synthesize results from the set of long-term experiments conducted in a wide variety of ecosystems to demonstrate consistent impacts of fragmentation, how those impacts change over time, and how they align with predictions from theory and observation. Finally, we identify key knowledge gaps for the next generation of fragmentation experiments.

## GLOBAL ANALYSIS OF THE EXTREME MAGNITUDE AND EXTENT OF FRAGMENTATION

New satellite data sets reveal at high resolution how human activities are transforming global ecosystems. Foremost among these observations are those of forest cover because of the high contrast between forest and anthropogenic land cover types. Deforestation, which was already widespread in temperate regions in the mid-18th to 20th centuries and increased in the tropics over the past half century, has resulted in the loss of more than a third of all forest cover worldwide (*10*, *11*). Beyond the direct impacts of forest loss and expanding anthropogenic land cover (for example, agricultural fields and urban areas), remnant forests are likely to suffer from being smaller, more isolated, and with a greater area located near the edge of the forest (*12*).

**Fig. 1:**
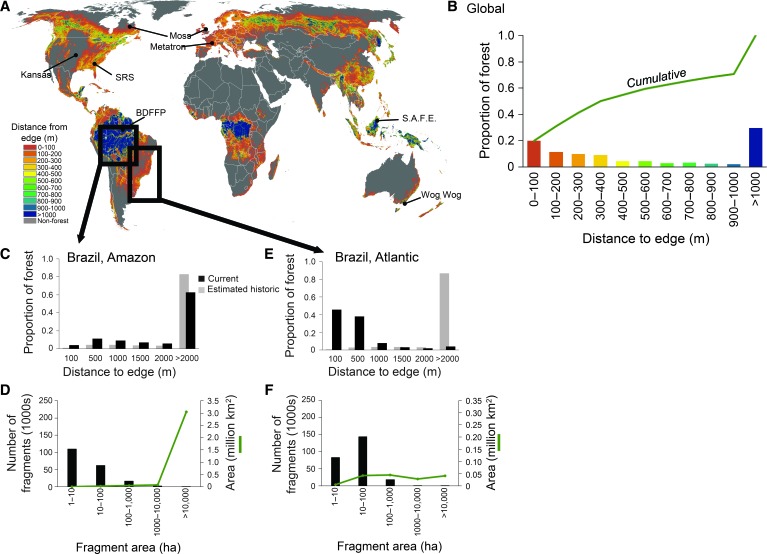
The global magnitude of forest fragmentation. (**A**) Mean distance to forest edge for forested pixels within each 1-km cell. Lines point to locations of ongoing fragmentation experiments identified and described in Fig. 2. (**B**) Proportion of the world’s forest at each distance to the forest edge and the cumulative proportion across increasing distance categories (green line). (**C** and **E**) In the Brazilian Amazon (C) and Atlantic Forests (E), the proportion of forest area at each distance to forest edge for both the current and estimated historic extent of forest. (**D** and **F**) In the Brazilian Amazon (D) and Atlantic Forests (F), the number of fragments and the total area of fragments of that size. The total number of fragments in the smallest bin (1 to 10 ha) is an underestimate in both the Atlantic Forest and Amazon data sets because not all of the very smallest fragments are mapped.

We analyzed the world’s first high-resolution map of global tree cover (*13*) to measure the magnitude of forest fragmentation. This analysis revealed that nearly 20% of the world’s remaining forest is within 100 m of an edge (Fig. 1, A and B)—in close proximity to agricultural, urban, or other modified environments where impacts on forest ecosystems are most severe (*14*). More than 70% of the world’s forests are within 1 km of a forest edge. Thus, most forests are well within the range where human activities, altered microclimate, and nonforest species may influence and degrade forest ecosystems (*15*). The largest contiguous expanses of remaining forests are in the humid tropical regions of the Amazon and Congo River Basins (Fig. 1A). Large areas of more disjunct forest also remain in southeastern Asia, New Guinea, and the boreal biomes.

Historical data enable the study of the process of forest fragmentation over time. We reconstructed the historical forest extent and timing of fragmentation in two forested regions of Brazil that provide a stark contrast in land-use dynamics. The Brazilian Amazon is a rapidly changing frontier (*10*), yet most of its forests remain contiguous and far from an edge despite recent increases in fragmentation (Fig. 1, C and D). In contrast, the Brazilian Atlantic Forest is a largely deforested landscape, cleared for agriculture and logged for timber over the last three centuries (*11*). This remaining forest is dominated by small fragments, with most fragments smaller than 1000 ha and within 1000 m of a forest edge (Fig. 1, E and F) (*16*). In the Brazilian Amazon, the proportion of forest farther than 1 km from the forest edge has decreased from 90% (historical) to 75% (today), and in the Brazilian Atlantic, from 90% to less than 9%.

These two forested regions of Brazil define extremes of the fragmentation process and are representative of the extent of fragmentation in forested landscapes worldwide (Fig. 1), as well as many other biomes including temperate grasslands, savannas, and even aquatic systems (*17*). For example, although a spatial analysis similar to that of forest is not currently possible in grasslands, 37% of the world’s grassland eco-regions are classified as “highly fragmented” (*18*, *19*). Robust knowledge of how habitat fragmentation affects biodiversity and ecosystem processes is needed if we are to comprehend adequately the implications of this global environmental change.

## THE VALUE OF LONG-TERM FRAGMENTATION EXPERIMENTS

Long-term experiments are a powerful tool for understanding the ecological consequences of fragmentation (*20*). Whereas observational studies of fragmented landscapes have yielded important insights (*9*, *21*), they typically lack rigorous controls, replication, randomization, or baseline data. Observational studies have limited ability to isolate the effects of fragmentation from concomitant habitat loss and degradation per se (*4*, *7*, *22*). Remnant fragments are embedded in different types and qualities of surrounding habitat, complicating interpretation because the surrounding habitat also influences biodiversity and ecosystem productivity (*23*).

**Fig. 2:**
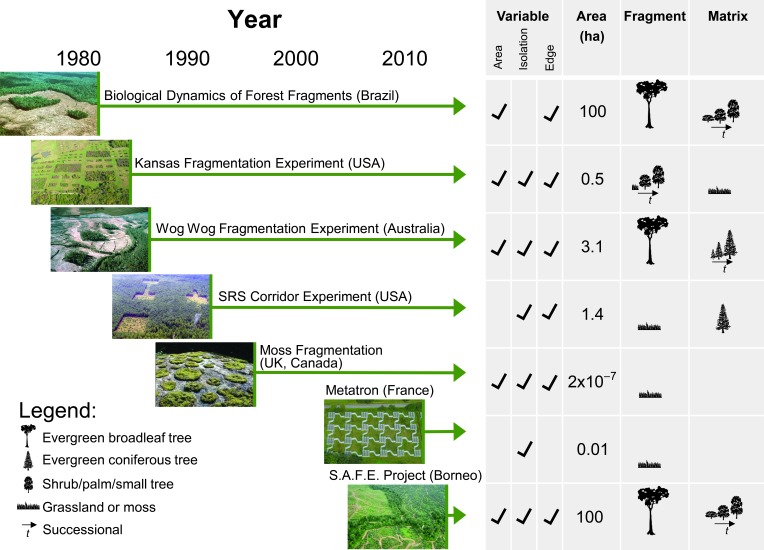
The world’s ongoing fragmentation experiments. All experiments have been running continuously since the time indicated by the start of the associated arrow (with the exception of the moss fragmentation experiment, which represents a series of studies over nearly two decades). The variables under study in each experiment are checked. The area is that of the experiment’s largest fragments. Icons under “Fragment” and “Matrix” indicate the dominant community and its relative height, with multiple trees representing succession.

The long-term fragmentation experiments we analyze here comprise the entire set of ongoing terrestrial long-term experiments. They occur in several biomes (Fig. 2 and Supplementary Materials) and were designed to manipulate specific components of fragmentation—habitat size, isolation, and connectivity—while controlling for confounding factors such as the amount of habitat lost across a landscape (Fig. 2). The largest fragments across these experiments match the size of fragments commonly created by anthropogenic activities (Figs. 1 and 2). Distances to the edge of experimental fragments range to 500 m, encompassing edge distances found in more than half of forests worldwide (Fig. 1B). In each experiment, different fragmentation treatments with replication were established, starting from continuous, nonfragmented landscapes and controlling for background environmental variation either by experimental design (blocking) or by measurement of covariates for use in subsequent analyses. Tests were conducted within fragments that varied experimentally in area or edge, within fragments that were experimentally isolated or connected, or within experimental fragments compared to the same area within continuous habitat. All treatments were replicated. Experiments were created by destroying or creating precise amounts of habitat across replicate landscapes, allowing tests of fragmentation effects independent of habitat loss. The robust and comparable experimental designs allow for powerful tests of the mechanisms underpinning the ecological impacts of fragmentation, and the long-term nature of ensuing studies has revealed consistent emergent effects.

**Fig. 3:**
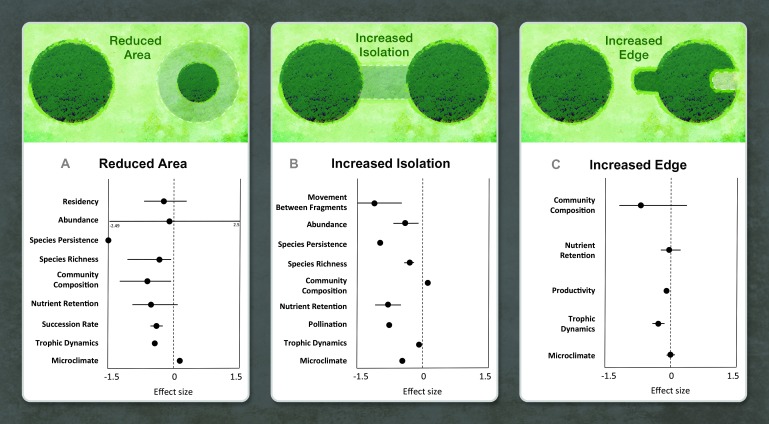
Fragmentation effects propagate through the whole ecosystem. (**A** to **C**) For each fragmentation treatment [reduced area in BDFFP, Wog Wog, Kansas (A); increased isolation in SRS and Moss (B); and increased edge in all experiments (C)], we summarize major findings for ecological processes at all levels of ecological organization. Each dot represents the mean effect size [computed as log response ratio: ln(mean in more fragmented treatment/mean in non- or less-fragmented treatment)] for an ecological process. Effect sizes are statistical, such that negative or positive values could represent degrading function. Horizontal bars are the range when a dot is represented by more than one study. Details, including individual effect sizes for each study, are reported in table S1.

These experiments mimic anthropogenic fragmentation; they are whole-ecosystem manipulations in which all species and processes experienced the same treatment (*24*). Emergent responses thus reflect the multiple direct and indirect effects of interacting species and processes. Further, because experimentally fragmented ecosystems are open to fluxes of individuals and resources, fragmentation effects can manifest across multiple levels of ecological organization (Fig. 3). Long-term experiments have the power to detect lagged and/or chronic impacts.

The first fragmentation experiments, now more than three decades old, were created to test effects of fragment area on both species persistence and patterns of immigration, reflecting concern in conservation biology about the role of fragmentation in reducing population sizes below viable levels (*25*) (Fig. 2). Subsequent experiments, created two decades ago, shifted focus to modifying habitat isolation, reflecting recognition of the potential to mitigate negative effects of fragmentation by recreating habitat—specifically with corridors—to increase connectivity among fragments (*26*) (Fig. 2). The newest experiments test emerging questions about potentially deleterious synergies between fragmentation and global changes in climate and land use (Fig. 2).

We synthesized results available 31 January 2014 for all studies within these experiments that were conducted in all treatments and replicates, and tested fragmentation effects on dispersal, abundance, extinction, species richness, community composition, and ecosystem functioning. We first calculated effect sizes of fragmentation as log response ratios (Fig. 3). Data from 76 different studies across the five longest-running experiments were drawn from published and unpublished sources (table S1). We synthesized results according to three fragmentation treatments: reduced fragment area [the focus of Biological Dynamics of Forest Fragments Project (BDFFP), Wog Wog, and Kansas; see Fig. 2 for identifiers of experiments], increased fragment isolation [Savannah River Site (SRS) and Moss], and increased proportion of edge (all experiments). Fragmented treatments were compared directly to non- or less-fragmented habitats that were either larger or connected via structural corridors (table S1).

### Strong, consistent, and accumulating effects of habitat fragmentation

Our synthesis revealed strong and consistent responses of organisms and ecosystem processes to fragmentation arising from decreased fragment area, increased isolation, and the creation of habitat edges (Fig. 3).

Community and ecosystem responses emerge from observed responses at the level of populations. Reduced area decreased animal residency within fragments, and increased isolation reduced movement among fragments, thus reducing fragment recolonization after local extinction (Fig. 3, A and B). Reduced fragment area and increased fragment isolation generally reduced abundance of birds, mammals, insects, and plants (Fig. 3, A and B). This overall pattern emerged despite complex patterns of increases or declines in abundance of individual species (Fig. 3A) with various proximate causes such as release from competition or predation, shifts in disturbance regimes, or alteration of abiotic factors (*14*, *27*–*29*). Reduced area, increased isolation, and increased proportion of edge habitat reduced seed predation and herbivory, whereas increased proportion of edge caused higher fledgling predation that had the effect of reducing bird fecundity (represented together as trophic dynamics in Fig. 3, A to C). Perhaps because of reduced movement and abundance, the ability of species to persist was lower in smaller and more isolated fragments (Fig. 3, A and B).

As predicted by theory (*6*, *30*, *31*), fragmentation strongly reduced species richness of plants and animals across experiments (Fig. 3, A and B), often changing the composition of entire communities (Fig. 3, A to C). In tropical forests, reduced fragment size and increased proportion of edge habitat caused shifts in the physical environment that led to the loss of large and old trees in favor of pioneer trees (Fig. 3, A and C), with subsequent impacts on the community composition of insects (*32*). In grasslands, fragment size also affected succession rate, such that increased light penetration and altered seed pools in smaller fragments impeded the rate of ecological succession relative to that of larger fragments (*33*) (Fig. 3A).

Consistently, all aspects of fragmentation—reduced fragment area, increased isolation, and increased edge—had degrading effects on a disparate set of core ecosystem functions. Degraded functions included reduced carbon and nitrogen retention (Fig. 3, A to C), productivity (Fig. 3C), and pollination (Fig. 3B).

In summary, across experiments spanning numerous studies and ecosystems, fragmentation consistently degraded ecosystems, reducing species persistence, species richness, nutrient retention, trophic dynamics, and, in more isolated fragments, movement.

### Long-term consequences of fragmentation

**Fig. 4:**
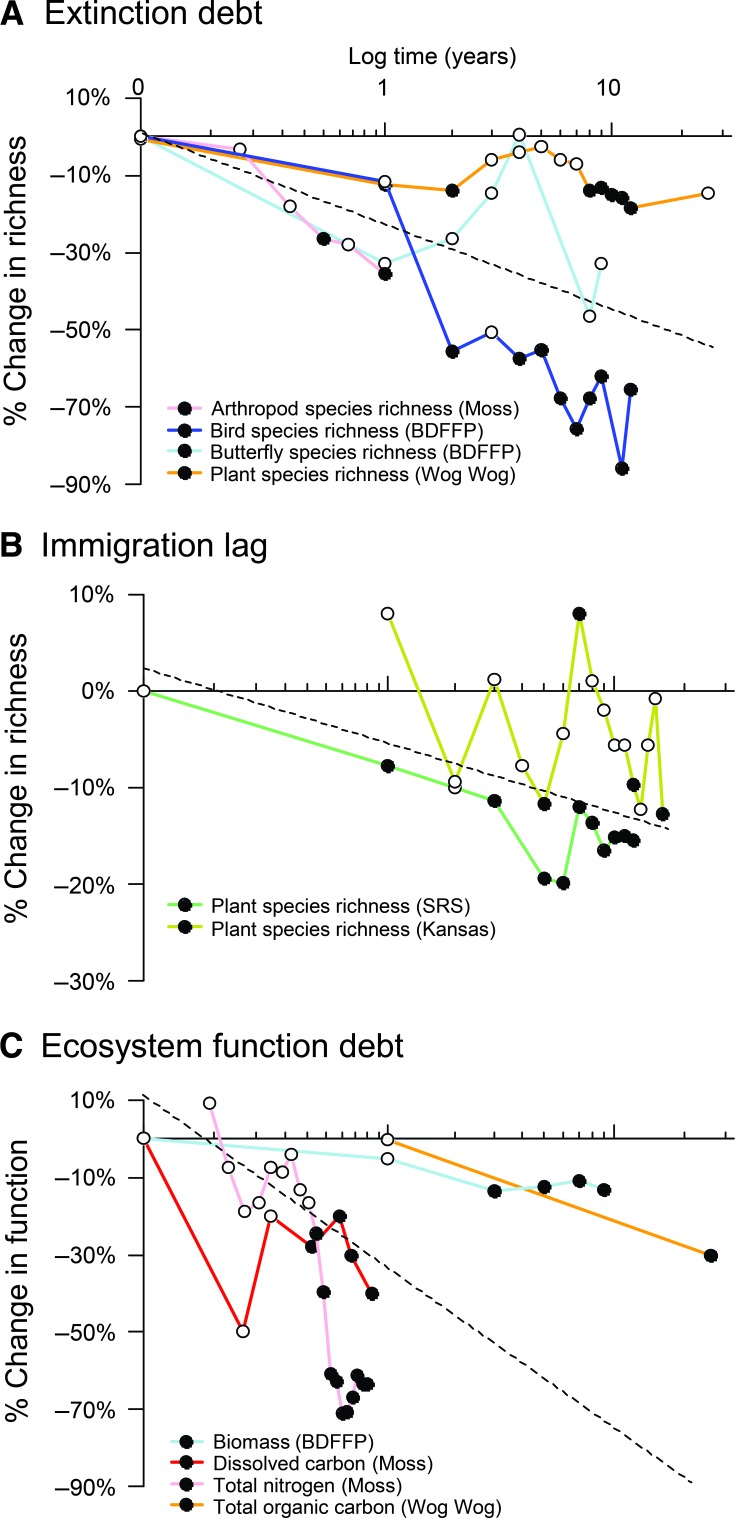
Delayed effects of fragmentation on ecosystem degradation. (**A**) The extinction debt represents a delayed loss of species due to fragmentation. (**B**) The immigration lag represents differences in species richness caused by smaller fragment area or increased isolation during fragment succession. (**C**) The ecosystem function debt represents delayed changes in ecosystem function due to reduced fragment size or increased isolation. Percent loss is calculated as proportional change in fragmented treatments [for example, (no. of species in fragment − no. of species in control)/(no. of species in control) × 100]. Fragments and controls were either the same area before and after fragmentation, fragments compared to unfragmented controls, or small compared to large fragments. Filled symbols indicate times when fragmentation effects became significant, as determined by the original studies (see table S2). Mean slopes (dashed lines) were estimated using linear mixed (random slopes) models. Mean slope estimates (mean and SE) were as follows: (A) −0.22935 (0.07529); (B) −0.06519 (0.03495); (C) −0.38568 (0.16010).

To synthesize all time series of species richness and ecosystem functioning gathered across experiments, we measured effects of fragmentation over the course of each study. The effect of fragmentation was calculated over time as the proportional change in fragmented relative to non- or less-fragmented treatments (Fig. 4).

In most cases, the large and consistent effects of fragmentation revealed by the experiments were predicted from theory. However, we were struck by the persistence of degradation to biodiversity and ecosystem processes and by the increase in many of the effects over time (Fig. 4). For example, extreme rainfall events at Wog Wog appeared to delay the decline in plant species richness for 5 years after fragmentation. In the Kansas Experiment, a lag of 12 years occurred before fragmentation effects on plant succession were detected. Our results thus reveal long-term and progressive effects of fragmentation and provide support for three processes proposed by recent studies in spatial ecology: extinction debt, immigration lag, and ecosystem function debt (Fig. 4).

First, we found strong evidence for temporal lags in extinction [that is, “extinction debt” (*30*)] in fragments. Species richness of plants, arthropods, and birds sampled in the experiments conducted in mature forest fragments and replicated moss landscapes showed decreases of 20 to 75% after fragmentation (Fig. 4A). Some declines were evident almost immediately after fragmentation, whereas others increased in magnitude over the experiment’s duration. Across experiments, average loss was >20% after 1 year, >50% after 10 years, and is still increasing in the longest time series measured (more than two decades). The rate of change appears to be slower in larger fragments [in BDFFP, 50% decline in bird species after 5 years in 1-ha fragments, but after 12 years in 100-ha fragments; in Moss, 40% decline in arthropod species richness of small fragments and 26% reduction in large fragments after 1 year (*34*, *35*)]. As predicted by theory (*36*), the extinction debt appears to take longer to pay in larger fragments.

Second, we observed that reduced richness was coincident with an “immigration lag” (*37*), whereby small or isolated fragments are slower to accumulate species during community assembly (*33*, *38*) (Fig. 4B). Immigration lags were observed in experiments conducted in successional systems that were initiated by creating new habitat fragments, rather than by fragmenting existing habitats. After more than a decade, immigration lags resulted in 5% fewer species after 1 year, and 15% fewer species after 10 years in small or isolated fragments compared to large or connected fragments (Fig. 4B).

Third, we observed an ecosystem function debt caused by fragmentation (*39*) in forest and moss fragments (Fig. 4C). An ecosystem function debt is manifest both as delayed changes in nutrient cycling and as changes to plant and consumer biomass. Loss of function amounted to 30% after 1 year, rising to 80% after a decade in small and isolated fragments when compared to larger and more connected fragments (Fig. 4C). Functional debts can result from biodiversity loss, as when loss of nutrients and reduction in decomposition are caused by simplification of food webs. Alternatively, the impact is exhibited through pathways whereby fragmentation changes biotic (for example, tree density in successional systems) or abiotic conditions (for example, light regimes or humidity) in ways that alter and potentially impair ecosystem function [for example, biomass collapse in fragments; Figs. 3 and 4; altered nitrogen and carbon soil dynamics (*40*)].

### A new understanding of the effects of fragmentation

By testing existing theory, experiments play a pivotal role in advancing ideas and developing new theory. We draw on experimental evidence to highlight two ways that the understanding of fragmentation has been enriched by the interplay between long-term experiments and development of theory.

First, island biogeography (*6*) was among the earliest theories to predict extinction and immigration rates and patterns of species richness in isolated biotas, which were later used to predict the effects of fragmentation on these variables. Experiments in continental settings tested the theory and gave rise to fresh perspectives. For example, islands are surrounded by sea, a thoroughly inimical matrix for island-dwelling species. Habitat islands, or fragments, are surrounded by a matrix that may not be so unsuitable for some species. In terms of all of the ecological variables studied in our long-term experiments, our results support the conclusion that ecological dynamics in human-modified fragments are a stark contrast to the dynamics in intact habitats that remain. Observational studies that have devoted more detailed consideration to the countryside within which fragments are embedded explain the diversity of ecological responses in remaining fragments (*41*). At the same time as experiments supported the core predictions of classical theories about effects of fragment size and isolation (Figs. 3 and 4), they spurred and tested new theories such as metacommunity theory (*42*) to account for variation in connectivity and habitat quality within and between fragments (*33*, *43*–*45*), spatial dynamics (*14*, *46*), and spatially varying interspecific interactions (*47*).

Second, experiments have demonstrated that the effects of fragmentation are mediated by variation in traits across species. More realistic predictions of community responses to fragmentation emerged after explicit consideration of species traits such as rarity and trophic levels (*48*, *49*), dispersal mode (*50*–*52*), reproductive mode and life span (*29*, *53*), diet (*54*), and movement behavior (*55*, *56*). Increasingly, the simple theoretical prediction that fragmentation reduces species richness is being modified to account for species identity through models that focus on how species vary in their traits (*4*, *21*, *36*, *48*, *57*, *58*). Consideration of traits may help to interpret variation around the overarching pattern that fragmentation consistently reduces species richness across many species and biomes (Figs. 3 and 4).

## A NEW GENERATION OF FRAGMENTATION EXPERIMENTS

New foci are emerging for studying ecosystem fragmentation, including (i) synergies between fragmentation and global changes, (ii) eco-evolutionary responses of species to fragmentation, and (iii) ecological responses to fragmentation in production landscapes—that is, ecosystems whose services are under extreme appropriation by humans (*59*).

First, conclusions from experiments thus far are likely to have been conservative because impacts from other environmental changes have been mostly excluded. Most forms of global change known to reduce population sizes and biodiversity will be exacerbated by fragmentation (*58*, *60*), including climate change (*61*), invasive species (*62*, *63*), hunting (*64*), pollution [including light, noise, and chemicals (*65*)], and altered disturbance regimes (*66*).

More complex experiments with unparalleled control and capacity to simultaneously manipulate fragmentation and other global changes are now under way (*53*). The Metatron, created in 2011 in southern France (*67*), enables ecologists to assess effects of variation in temperature and other abiotic factors in addition to habitat isolation. The SAFE Project is being created in the rainforest of Borneo (*68*) and will embed a fragmentation experiment within a production agricultural plantation in which poaching will occur. Other synergies should be investigated experimentally, including the interaction between fragmentation and hunting, fire, infectious disease outbreaks, or nitrogen deposition. Within these experiments, fragmentation and loss of habitat can then be varied independently.

Second, current experiments have stopped short of examining how fragmentation drives evolution through genetic bottlenecks, ecological traps, changing patterns of selection, inbreeding, drift, and gene flow (*69*–*72*). Extensive fragmentation has occurred over many years, and in some regions over millennia (*11*). Changes caused by fragmentation undoubtedly lead to altered patterns of selection and trait evolution. Evolutionary responses to fragmentation have already been suggested (*73*, *74*), and it is likely that such changes will, in turn, feed back to influence population persistence and ecosystem resilience in fragmented landscapes. Linking long-term experiments with the tools of landscape genetics (*75*) may provide powerful insights into the evolutionary dynamics of species inhabiting fragmented landscapes.

Third, new experiments should address the management of natural habitats in production landscapes by monitoring vegetation, networks of interacting species, and ecosystem services at ecologically relevant spatial and temporal scales (*76*–*78*). Some ecosystem services have global consequences, for example, local carbon sequestration affects global atmospheric CO_2_. However, in many cases the benefits obtained by people depend on their proximity to habitat fragments (*79*). For example, crop pollination and biological pest control from natural areas adjacent to farms are made available by the very process of habitat fragmentation, bringing people and agriculture closer to those services. Yet, further fragmentation reduces access to many services and ultimately may push landscapes past tipping points, beyond which essential ecosystem services are not merely diminished but lost completely (*80*). This complex relationship creates a double-edged sword, for which locally optimal levels and arrangements of habitat must be sought. New fragmentation experiments should consider how multiple fragments in a landscape interact, creating an ecological network in which the collective benefit of ecosystem services may be greater than the sum of services provided by individual fragments (*81*, *82*). Experimental inferences may then be tested beyond their spatiotemporal domains and, if successful, extrapolated across scales. Such research will be aided by satellite monitoring of ecosystems and human land use across the globe. The most powerful research programs will integrate experiments, observational studies, air- and space-borne imaging, and modeling.

## CONCLUSIONS

Fragmentation experiments—some of the largest and longest-running experiments in ecology—provide clear evidence of strong and typically degrading impacts of habitat fragmentation on biodiversity and ecological processes. The findings of these experiments extend to a large fraction of the terrestrial surface of the Earth. Much of the Earth’s remaining forest fragments are less than 10 ha in area, and half of the world’s forest is within 500 m of the forest edge—areas and distances matched to existing long-term experiments (Figs. 1 and 2) from which consistent effects of fragmentation have emerged (Figs. 3 and 4).

Reduced fragment area, increased isolation, and increased edge initiate changes that percolate through ecosystems (Fig. 3). Fragmentation has the capacity to generate persistent, deleterious, and often unpredicted outcomes, including surprising surges in abundance of some species and the pattern that long temporal scales are required to discern many strong system responses. In light of these conclusions and ongoing debates, we suggest that fragmentation’s consistency, pervasiveness, and long-term degrading effect on biodiversity and ecosystem function have not been fully appreciated (*9*).

Without gains in yield and efficiency of agricultural systems (*83*), the expansion of human populations will inevitably continue to reduce and fragment natural areas. The area of Earth’s land surface devoted to cropland already occupies 1.53 billion hectares (*83*) and may expand 18% by the middle of this century (*84*), and the area committed to urban centers is predicted to triple to 0.18 billion hectares by 2030 (*85*). The capacity of the surviving forests and other natural habitats to sustain biodiversity and ecosystem services will hinge upon the total amount and quality of habitat left in fragments, their degree of connectivity, and how they are affected by other human-induced perturbations such as climate change and invasive species. Long-term experiments will be even more needed to appreciate, explain, and predict long-term effects. New efforts should work in concert, coordinating a network of experiments across ecosystems and spatial extents.

The effects of current fragmentation will continue to emerge for decades. Extinction debts are likely to come due, although the counteracting immigration debts may never fully be paid. Indeed, the experiments here reveal ongoing losses of biodiversity and ecosystem functioning two decades or longer after fragmentation occurred. Understanding the relationship between transient and long-term dynamics is a substantial challenge that ecologists must tackle, and fragmentation experiments will be central for relating observation to theory.

Experimental results to date show that the effects of fragmentation are strong and markedly consistent across a diverse array of terrestrial systems on five continents. Increasingly, these effects will march in concert with other global changes. New experiments should be coupled with emerging technologies, landscape genetics, and detailed imagery of our planet, and should be coordinated with current ecological theory to understand more deeply the coupled dynamics of ecological and social systems. These insights will be increasingly critical for those responsible for managing and prioritizing areas for preservation and ecological restoration in fragmented landscapes.

## Supplementary Material

http://advances.sciencemag.org/cgi/content/full/1/2/e1500052/DC1

## SUPPLEMENTARY MATERIALS

Supplementary material for this article is available at http://advances.sciencemag.org/cgi/content/full/1/2/e1500052/DC1 Materials and Methods Fig. S1. Map of the BDFFP experiment and location within Brazil. Fig. S2. Map of the Kansas fragmentation experiment. Fig. S3. Map of the Wog Wog experiment and location within Australia. Fig. S4. Map of the SRS experiment showing locations of the eight blocks in the second SRS Corridor Experiment within the SRS, South Carolina, USA. Fig. S5. Design of the Moss experiment. Fig. S6. Design of the Metatron experiment with 48 enclosed fragments and adjoining enclosed corridors. Fig. S7. Map of the SAFE experiment and location within Borneo [after Ewers *et al*. (*68*)]. Table S1. Metadata for Fig. 3 in the main text. Table S2. Metadata for Fig. 4 in the main text.
