# Supplementary material for: Habitat fragmentation and its lasting impact on Earth’s ecosystems
Source: Sci Adv. 2015 Mar 20;1(2):e1500052. doi: 10.1126/sciadv.1500052 (PMC4643828; doi:10.1126/sciadv.1500052)
Supplement: http://advances.sciencemag.org/cgi/content/full/1/2/e1500052/DC1 [file supp_1_2_e1500052__index.html]

Science Advances | Science Advances

## Supplementary Materials

**This PDF file includes:**

- Materials and Methods
- Fig. S1. Map of the BDFFP experiment and location within Brazil.
- Fig. S2. Map of the Kansas fragmentation experiment.
- Fig. S3. Map of the Wog Wog experiment and location within Australia.
- Fig. S4. Map of the SRS experiment showing locations of the eight blocks in the second SRS Corridor Experiment within the SRS, South Carolina, USA.
- Fig. S5. Design of the Moss experiment.
- Fig. S6. Design of the Metatron experiment with 48 enclosed fragments and adjoining enclosed corridors.
- Fig. S7. Map of the SAFE experiment and location within Borneo after Ewers *et al*. (*68*).
- Table S1. Metadata for Fig. 3 in the main text.
- Table S2. Metadata for Fig. 4 in the main text.

Download PDF

**Files in this Data Supplement:**

- Adobe PDF - e1500052\_SM.pdf
