# Supplementary material for: Habitat fragmentation and its lasting impact on Earth’s ecosystems
Source: Sci Adv. 2015 Mar 20;1(2):e1500052. doi: 10.1126/sciadv.1500052 (PMC4643828; doi:10.1126/sciadv.1500052)
Supplement: http://advances.sciencemag.org/cgi/content/full/1/2/e1500052/DC1 [file e1500052_SM.pdf]

Science Advances

www.advances.sciencemag.org/cgi/content/full/1/2/e1500052/DC1

Supplementary Materials for

Habitat fragmentation and its lasting impact on Earth's ecosystems

Nick M. Haddad,* Lars A. Brudvig, Jean Clobert, Kendi F. Davies, Andrew Gonzalez, Robert D. Holt, Thomas E. Lovejoy, Joseph O. Sexton, Mike P. Austin, Cathy D. Collins, William M. Cook, Ellen I. Damschen, Robert M. Ewers, Bryan L. Foster, Clinton N. Jenkins, Andrew J. King, William F. Laurance, Douglas J. Levey, Chris R. Margules, Brett A. Melbourne, A. O. Nicholls, John L. Orrock, Dan-Xia Song, John R. Townshend

Published 20 March 2015, *Sci. Adv.* **1**, e1500052 (2015)
DOI: 10.1126/sciadv.1500052

This PDF file includes:

Materials and Methods
Fig. S1. Map of the BDFFP experiment and location within Brazil.
Fig. S2. Map of the Kansas fragmentation experiment.
Fig. S3. Map of the Wog Wog experiment and location within Australia.
Fig. S4. Map of the SRS experiment showing locations of the eight blocks in the second SRS Corridor Experiment within the SRS, South Carolina, USA.
Fig. S5. Design of the Moss experiment.
Fig. S6. Design of the Metatron experiment with 48 enclosed fragments and adjoining enclosed corridors.
Fig. S7. Map of the SAFE experiment and location within Borneo [after Ewers *et al.* (*68*)].
Table S1. Metadata for Fig. 3 in the main text.
Table S2. Metadata for Fig. 4 in the main text.

<u>Haddad et al. Supplementary Materials</u>

Materials and Methods

Brief Descriptions of the Habitat Fragmentation Experiments

<u>The Biological Dynamics of Forest Fragments Project (BDFFP)</u> (28º 30' S, 60º W; Supplementary Figure S1) is the world's largest and longest-running experimental study of habitat fragmentation. A full description of this experiment is provided by Laurance et al. (2011). It is located in central Amazonia, 70 km north of Manaus, Brazil. The study area, which spans about 1000 km^2 and ranges from 60-140 m elevation, was originally mostly dense, non-flooded (terra-firme) rain forest, dissected by numerous streams and gullies. Except for the experimental fragmentation, the site is largely free of anthropogenic disturbances such as selective logging, fires, and past agriculture. The forests are among the most species diverse in the world, with a typical canopy height of 37-40 m. The BDFFP largely occupies heavily-weathered, nutrient-poor soils. Rainfall ranges from 1900-3500mm annually, with a moderately strong dry season.
The study area is comprised of three large cattle ranches (3000-5000 ha each) containing 11 forest fragments (five of 1 ha, four of 10 ha, two of 100 ha). Expanses of nearby continuous forest serve as experimental controls. In the early- to mid-1980s, the fragments were isolated from nearby intact forest by distances of 80-650 m by clearing and often burning the surrounding forest. Pre-fragmentation censuses were conducted for trees and many faunal groups, allowing long-term changes in these groups to be confidently assessed. Because of poor soils and low productivity, the ranches surrounding the BDFFP fragments have been gradually abandoned. Secondary forests have since proliferated in many formerly cleared areas. To help maintain some isolation of the experimental fragments, 100 m-wide strips of regrowth were cleared and burned around each fragment on 3-4 occasions, most recently between 1999 and 2001. The 100 m-wide strips are currently being re-cleared around most of the BDFFP fragments.

<u>Kansas Fragmentation Experiment</u> is located at the University of Kansas Field Station, near Lawrence, KS, USA (39º 3' N, 95º 12' W; Supplementary Figure S2). Fragments of three sizes were created in 1984 on abandoned cropland: small fragments (4 x 8 m), medium fragments (8 x 12 m), and large fragments (50 x 100 m), as described in Holt et al. (1995). The matrix surrounding fragments has been mowed regularly since the inception of the experiment, while the fragments have undergone succession to the present day. To ensure adequate replication, only large (n=6) and small (n=6 clusters of 82 small fragments) fragments are used for analyses in this paper. A cluster of small fragments occupies the same area as one large fragment (0.5 ha). 30 sampling locations are equally spaced among small fragments in a cluster or one large fragment. Thus connectivity varied among sampling locations within a collection of small fragments separated by a mowed matrix, compared to among sampling locations within a large fragment. See Methods for Unpublished Studies – Kansas (below), for additional details.

<u>The Wog Wog Habitat Fragmentation Experiment</u> is located in southeastern New South Wales, Australia (37º 04' S, 149º 28' E; Supplementary Figure S3) in native sclerophyllous *Eucalyptus* forest. It is named for nearby Mt. Wog Wog. The experimental design and the rationale for it are provided in Margules (1992). It consists of three fragment sizes: 0.25 ha, 0.875 ha, and 3.062 ha. Four replicates of each size, 12 in total, became habitat fragments when the surrounding *Eucalyptus* forest was cleared in 1987 and planted to *Pinus radiata*, for plantation timber. The matrix surrounding fragments is thus composed of pine plantation (*P. radiata*) that is commercially managed forest. Between 1987 and the present, pines in the matrix have grown from seedlings to mature trees that are now slightly taller than the native eucalpyt forest, and the pine canopy is now mostly closed. Two replicates of each size, six in total, serve as the unfragmented controls in uncleared continuous forest. Within fragments, sampling is stratified in two ways: first, by habitat type into slopes and drainage lines because the vegetation communities associated with these topographic features are different (Austin and Nicholls 1988). Slopes are characterized by a grassy understory and scattered shrubs below open *Eucalyptus* forest. Drainage lines are dominated by *Kunzea*, a small shrubby tree that forms dense stands. Second, sampling is stratified by proximity to the fragment edge (edge or interior). There are two monitoring sites in each of the four strata (slope edge, slope interior, drainage-line edge, drainage-line interior), totaling eight sample sites within each fragment for a total of 144 sites over the 18 fragments (Davies and Margules 1998). Following matrix clearing in

1987, an additional 44 monitoring sites were established in the matrix between the habitat fragments, also stratified by habitat type. Two permanent pitfall traps, and a permanent herbaceous-vegetation plot are located at each of the 188 monitoring sites. Arthropods and some small vertebrates such as skinks and frogs are collected in the pitfall traps. Monitoring commenced in 1985 and two years of data were collected before the fragmentation treatment was applied in 1987. Monitoring then continued through 2000 for animals, and until 1998 for plants. Vegetation plots on slopes were resampled in 2010. Monitoring recommenced in 2009 for invertebrates, and has continued to the present. See Methods for Unpublished Studies – Wog Wog (below), for additional details.

Savannah River Site (SRS) Corridor Experiment is located at the Savannah River Site, a National Environmental Research Park near New Ellenton, South Carolina, USA (33° 20' N, 81° 40' W; Supplementary Figure S4). The results in this paper draw from two different experiments, the first occurred from 1993-2000 described in Haddad (1999) and the second from 2000-present described in Tewksbury et al. (2002). All fragments were created by clearing pine trees within a large plantation of *Pinus palustris* and *P. taeda* trees ~22m in height, which now forms the matrix surrounding fragments. Fragments are open habitats dominated by herbs and shrubs, succeeding toward longleaf pine savanna over time and maintained with hardwood removal and prescribed fire every ~2-3 years. In the first experiment, the 27 fragments were each 128 x 128 m. Some fragments were isolated and others were connected by 32 m wide corridors ranging from 64-384 m in length. In the second experiment, which constitutes the bulk of studies and the longest time series, 40 fragments are arranged in 8 blocks. Blocks are separated from each other by 1-20 km. Within each block, a central 100 x 100 m (1 ha) fragment is surrounded by four other fragments, one of which is connected by a 150 m long and 25 m wide corridor. The other three unconnected fragments vary in shape based on two treatments. One treatment was created by adding an area equal to that of the corridor to the fragment, creating a rectangular fragment of 1.375 ha. The other treatment was created by adding both the area and shape of the corridor, creating a "winged" fragment of 1.375 ha with two, 75 x 25 m "wings" projecting from opposite sides of the patch. See Methods for Unpublished Studies – SRS (below), for additional details

The Moss Fragmentation Experiments were conducted in the field in the UK and Canada, and in the lab in a growth chamber at the University of Nottingham (UK).

1.1 Fragmentation experiment: This and the corridor experiment described below were conducted in the Derbyshire Peak District, northern England UK (53° 08' N, 1° 57' W). In October 1995, two treatments were established, control and fragmented, in a randomized block design using eight moss-covered boulders (mainly *Hypnum cupressiforme*, *Thuidium tamariscinum,* and *Tortella tortuosa*) described in Gilbert et al. (1998). Each replicate boulder contained 12 randomly distributed circular moss fragments, six 20 cm^2 and six 200 cm^2, and a continuous moss carpet acting as an undisturbed control (minimum area: 50 × 50 cm). The fragmented treatment was created using a template to ensure constancy in fragment area and distance (15cm) between adjacent fragments. Habitat fragments were created on one half of the boulder by scraping and removing the moss cover; these moss fragments were left surrounded by bare rock for the entire duration of the experiment, a habitat considered inhospitable for most mite taxa. Community responses to fragmentation were monitored over a 12-month period encompassing several generations for the larger predatory mites (equivalent to several generations for many of their prey species). Every 2 months, one moss fragment was chosen randomly and removed from each block. Moss samples of equal area were also removed from the control treatment on each sampling date. This control allowed for seasonal changes in species abundance and diversity.

1.2 Corridor experiment (Supplementary Figure S5): In October 1995, four experimental fragmentation treatments were established on moss-covered boulders, each consisting of four circular fragments of moss 10 cm in diameter described in Gilbert et al. (1998). Fragment centers were placed at the corners of a square of side 17 cm (i.e., fragments were separated 7 cm from each other); treatments were at least 10 cm apart on each rock, and at least 10 cm from the remaining 'mainland' of moss. Fragmentation treatments were: (1) mainland (four circular samples, 10 cm diameter taken from the surrounding matrix of continuous moss), (2) corridor (four fragments connected along the sides of the square by corridors 7 cm long by 1cm wide), (3) broken corridor (like the corridor treatment, but corridors split in the middle and separated by a gap of 5 cm to provide a control for the increased area of the corridor treatment), and (4)

isolated (fragmented, but no corridors present). Thus, there were four replicate islands per treatment, with all four treatments replicated on each of six rocks. The ‘inhospitable’ rock surface between moss islands is probably a partial, not absolute, barrier to mite movement

2. Fragmentation and climate change experiment: initiated in June 2007 at a site in the subarctic-boreal forest region near the town of Schefferville, in northern Quebec, Canada (54°48′ N, 66°49′ W) (described in Lindo et al. 2012). The experimental area was composed of eight replicate sites (blocks) within a 2.4 hectare area. Site development and sampling occurred in contiguous areas of *Pleurozium schreberi* moss. Within each site, replicate plots were created on the forest floor in 2007 for destructive sampling in 2008 and 2009. Plots consisted of individual patches of *P. schreberi* that were either contained within 115 cm wide at the base, 69cm across the top and 40 cm tall, hexagonal open-topped chambers (OTC) or left under ambient conditions. Within the chamber treatments, the OTCs created a strong moisture gradient by acting as a rain shadow at the periphery of the chamber (effectively 25 cm wide) while the area in the middle of the OTC received precipitation similar to ambient conditions. The effect of the OTC increased the temperature at the soil surface by an average of 0.5 °C over the year, driven mainly by a 2° C increase in daily maxima during the summer months.

Moss patches (12.5 cm diameter and 9 cm deep) were cut from the surrounding matrix, placed in plastic plant pots, then exposed to one of four treatments: (1) outside of the OTC under ambient conditions (ambient), (2) within the inner area of the OTC (inner), (3) at the outer periphery of the OTC (outer), or (4) at the outer periphery of the OTC but open to the surrounding moss habitat by two, 3 cm wide openings on each side (corridors). The ambient moss patches served as a control to explore the effects of temperature, moisture, and ‘openness’ on community disassembly: differences between ambient and inner-chamber patches tested the effect of temperature (contrast 1), differences between inner-chamber patches and outer-chamber patches test the effect of drought (contrast 2) and differences between outer-chamber patches with and without corridors test the effect of openness in the presence of drought (contrast 3).

3. Lab Experiment: Experimental microcosms were created, consisting of four metacommunities of varying connectivity: (1) small island fragments with no corridors, (2) small fragments with broken corridors, (3) small fragments connected by corridors and (4) a large continuous habitat (described in Staddon et al. 2010). These treatments are similar to a design previously used (Gilbert et al. 1998, Gonzalez et al. 1998) in the field. Microcosms were constructed of 30 mm thick, 240 mm square PVC base with four 70 mm diameter subchambers in each corner (see Figure S1 of Staddon et al. 2010)). Each subchamber was 60 mm high and had a total volume of 0.23 L. Island microcosms consisted of only the four subchambers, whereas strips of moss 77 mm long and 17 mm wide were used to connect subchambers in the broken and corridor treatments. In the broken treatment, the strips were blocked in the middle with a 4 mm thick PVC divider.

Carpets of feather moss (*Thuidium tamariscinum*) and underlying detritus were collected on 12 January 2005 from the surface of large rocks in Derbyshire, England (53°6.4’ N, 1°36.4’ W). The moss was cut into circles of the same diameter as the subchambers, fresh weighed and placed in the subchambers. Strips of moss were cut to fill the connecting links in corridor and broken treatments. The continuous microcosms were similarly constructed on a 310 mm square base, but the main areas surrounding subchambers were filled with moss; thus these subchambers were not physically separated from the surrounding moss. The total volume of moss in each microcosm treatment was: continuous = 967.2 cm^3, corridor = 205.9 cm^3, broken = 203.1 cm^3, and island = 149.6 cm^3, but in all cases measurements were taken from subchamber-sized sections of the moss carpet.

Microcosms were allocated a 30 mm deep Perspex lid (3 mm thick), which fitted tightly along the contours of the subchambers and their corridors. Lids for the continuous microcosms, in addition to covering the subchambers, also covered the whole continuous area and included a 30 mm deep Perspex skirt around their edge. Drainage outputs (2 mm diameter) were located at the center of each subchamber, within the corridor strips, and regularly throughout the main area of the continuous microcosms. Each subchamber had an air inlet and outlet, fashioned from stainless steel pipe, with a 2 mm internal diameter. Ambient air from outside the laboratory was passed through a pre- filter (to remove particulates) and a 430 L buffer

chamber to dampen short-term fluctuations in CO_2 concentration, and humidified to minimize moss desiccation between watering and then delivered to the subchambers at 100 mL/min.
Each moss-filled experimental microcosm type was replicated five times, placed and maintained in a climate-controlled plant-growth room for 315 days (from 12 January to 23 November 2005). A fully randomized experimental design was used to eliminate any effect of lighting and airflow on the various treatments. In addition, a set of empty microcosms, one of each treatment type, was used to factor out any perturbation in measured CO_2 concentration values. Microcosms were maintained for the first 16 weeks of the experiment with a diurnal cycle set at 12⁄12 h (temperature 15°C ⁄ 12°C), after which the diurnal cycle was switched to 14⁄10 h (temperature 18°C⁄15°C) for the duration of the experiment. The photosynthetically active radiation (PAR) at moss height ranged from 400 to 450 µmol m^2/sec.

The Metatron was created in spring 2011 and is located in the south of France at Caumont (a small village 100 km south of Toulouse; 44° 27' N, 3° 44' W). The Metatron is described in detail in Legrand et al. 2012 and consists of a set of 48 (10 m x 10 m x 2.5 m) enclosures connected one to one by a 19 m long, double corridor (Supplementary Figure S6). Enclosures and corridors are covered by a net of 0.1 mm mesh size and isolated from the surrounding field by a 0.5 m tall plastic wall. A mobile roof can be deployed above each enclosure to reduce ground-level light by up to 80%. In the same way, the humidity can be increased up to 100% by the use of a sprinkler in the center of each enclosure. Temperature, luminosity, and humidity are recorded every 15 min. The corridors can be closed or open, allowing different "landscape" designs to be constructed (stepping stone, mainland-island model, etc.). The connections among enclosures can also be manipulated independently for ground-dwelling and flying species, allowing for species-specific exchanges to be altered within a meta-community. The ground layer within the enclosures and corridors is typically grassland, but can be modified. Current experiments concern the response of spatially structured populations to climate change for a lizard and the hierarchy among factors driving dispersal for a butterfly (Trochet et al. 2013).

The S.A.F.E. Project is located in the Malaysian state of Sabah on the island of Borneo (4°43' N, 117°36' W, Supplementary Figure S7). It consists of a gradient of forest disturbance encompassing primary rainforest, continuous logged rainforest, logged and experimentally fragmented rainforest in an oil palm plantation matrix, and continuous oil palm plantation described in Ewers et al. (2011). The experimental fragmentation is currently in process (initiated in 2013). Within the fragmented landscape there are two landscape design experiments. The first is the creation of six blocks of forest fragments, each containing one 100 ha, two 10 ha, and four 1 ha fragments. Fragments are aligned to allow an equal amount and spatial distribution of sampling in the three size classes of fragment. In addition, a 2200 ha Virgin Jungle Reserve will be isolated by the deforestation, creating a single, large fragment. Blocks are isolated from continuous forest by distances of 50 – 4,000 m and forest cover in the landscapes surrounding individual blocks will vary between 16-50%. The second landscape design experiment is creating riparian corridors along first-order streams with an approximate watershed area of 260 ha. Riparian corridors will be created with widths of 0, 15, 30 (the legal requirement in Malaysia), 60 and 120 m on either side of the permanent streams, and are matched with control streams in primary forest, logged forest and oil palm plantation. All fragments will be embedded in a working oil palm plantation in a landscape that will be initially deforested, terraced and then planted with oil palms that will take approximately eight years to form a closed canopy.

Fragmentation Analysis Methods

Fragmentation Analysis – Global
The global distance-to-edge map and histogram (Figure 1A, B) were generated from a global, 30-m resolution raster dataset of percent tree cover for the year 2000 (Sexton et al. 2013). Pixels covered with clouds or shadows in 2000 were filled with values from the same dataset in 2005, and those obscured by clouds or shadows in both 2000 and 2005 were filled with values from the MOderate-resolution Imaging Spectrometer (MODIS) Vegetation Continuous Fields (VCF) tree cover layer for the year 2000 (DiMiceli et al. 2011). Following the United Nations' International Geosphere-Biosphere Programme definition of forest (Belward 1996), tree-cover values were converted from percentages to binary forest/non-forest cover by applying a threshold of 30% cover: pixels with tree cover less than 30% were labeled "non-

forest", and those with tree cover greater than or equal to 30% were labeled "forest". A minimum mapping unit (MMU) filter was then applied to the binary map, re-coding the values of any contiguous group of pixels—whether forest or non-forest—whose combined area was less than one hectare to that of the surrounding pixels. The resulting 30-m resolution binary raster of forest vs. non-forest cover with MMU of 1 ha was then coarsened to 90-m resolution using a majority rule.

For each 90-m pixel labeled "forest", the horizontal Euclidean distance was calculated to the nearest "non-forest" pixel. Non-forest pixels were coded with null values. Histograms were constructed from these data for each continent and were summed globally. Distance from forest to nearest edge was mapped by resampling the values from 90-m to 1-km resolution, using bilinear interpolation. Because this process takes an (area-weighted) average of all forest pixels within the extent of each 1-km pixel, even 1-km pixels with only one 90-m forest pixel show a distance value in our projection (Figure 1A). Pixels—especially those with small edge-distances—should not be interpreted as fully forested.

Fragmentation Analysis – Brazil

For the analysis of forest fragmentation in Brazil (Figure 1C-F), two Landsat-based datasets from the Brazilian space agency (INPE) were used. Using these data, distributions of fragments of various sizes were calculated, an analysis that is not yet possible using the global scale forest data. For the Amazon, 2012 data were used from the PRODES deforestation monitoring program (Câmara et al. 2006). For the Atlantic Forest, the SOS Mata Atlântica/INPE dataset for 2005 was used, corresponding to the benchmark analyses in Ribeiro et al. (2009). The status of the Atlantic Forest has worsened slightly since 2005, with an estimated 1,500 km^2 having been lost since then (http://www.sosma.org.br), about 1% of the forest remaining in 2005.

Metrics of fragment size were derived using the original polygon versions of both datasets. To resolve the issue of contiguous forest polygons, a consequence of the original mapping methods to create the datasets, any boundaries shared between forest polygons were desolved. For the Atlantic Forest, forest types were not distinguished, retaining all forest categories as simply forest. Fragment sizes were calculated using an equal area map projection.

To estimate the original amount of forest near an edge for each biome, maps were first constructed of probable original forest extent. For the Amazon, Olson et al. (2001) ecoregions corresponding to the Amazon were used, clipped by the boundaries of the Legal Amazon, which corresponds to that part of the Amazon in Brazil. All areas were then marked in the PRODES dataset classified as historically non-forest (i.e., não floresta, hidrografia) as non-forest in the original forest extent. For the Atlantic Forest, the original extent is less certain because the forest was cut mostly decades or even centuries ago. The boundary was defined as by the Brazilian Institute of Geography (IBGE). Unlike the Amazon data, the map of estimated original Atlantic Forest did not include where rivers are even though major rivers do create edge in the Atlantic Forest. To make the maps more comparable, the HydroSHEDS dataset (Lehner et al. 2008) was used to cut major rivers into the original forest extent. Any stretch of river with 10,000 or more upstream cells was considered large enough to count as edge creators.

To calculate distance to edge, the Amazon data was first converted to a raster of 100-meter pixels and the Atlantic Forest data to a raster of 30 m pixels. Because the Amazon has a larger spatial extent, it was not feasible to rasterize it to the same resolution as the Atlantic Forest data. Distances were calculated as simple Euclidean distance using an equidistant map projection. Analyses were done using ArcMap 10.2.

Methods for Analysis of Figure 4

For the analysis to estimate mean slopes in Fig. 4 we used a linear mixed effects model with random slopes:

$$y_i = \alpha + \beta_{j[i]} x_i + \varepsilon_i$$
$$\beta_j \sim \text{Normal}(\beta, \sigma^2_\beta)$$
$$\varepsilon \sim \text{Normal}(0, \sigma^2)$$

where y is percent change, x is log(years), α is the intercept, β_j is the slope for study j, ε is the residual error, β is the mean slope (the parameter of interest), σ^2_β is the variance in slope among studies, σ^2 is the

residual variance, and *i* indexes the data points. We used the lmer function from the package lme4 (version 1.1-7) in R to fit this model.

Methods for Unpublished Studies

Methods for Unpublished Studies – Kansas

Soil temperature data: I-button temperature loggers (Embedded Data Systems) were embedded 5 cm under the soil surface in the center of 50 small (S) patches (one per patch), and 78 sites in large (L) patches (six edge sites and seven interior sites in each of six large patches). I-buttons recorded temperature every four hours from 16 July, 2012 to 22 June, 2013. Data were grouped according to season: Winter = Dec, Jan, Feb; Spring = Mar, Apr, May; Summer = June, July, Aug; Fall = Sep, Oct, Nov. In both summer and winter, significantly higher maximum temperatures were detected in small patches than in large patches (Patch size * Season: $F_{3,476}$=21.97, p<0.001 in two-way ANOVA, followed by Tukey Method for posthoc comparisons).

Beta-diversity: Permutational analysis of multivariate dispersions (PERMDISP, Anderson et al. 2006)) was employed using the PRIMER-E package (Clarke and Gorley 2006), to evaluate effects of habitat fragmentation on plant community dissimilarity. Homogeneity of within-group multivariate dispersions were evaluated between sampling units (1 m^2 sampling quadrats) embedded in small versus large patches based on the Bray-Curtis presence/absence coefficient. Separate analyses were conducted for each year in which a full dataset was available (1984-1990; 1994-2002). Fragmentation effects on community dissimilarity were dynamic over time. During the first 5 years of succession (1984-1988) mean community dissimilarity was reduced by fragmentation, significantly so (*P*<0.05) in 1984, 1986 and 1988. However, in all eleven subsequent sample years (1989 to 2002) over which woody plants increased in dominance, mean community similarity was increased by fragmentation, significantly so (*P*<0.05) in all sample years except 1995. Over this time period (1989-2002), the mean fragmentation effect (% increase in community dissimilarity) was 6.9% (mean *P*=0.02).

Methods for Unpublished Studies – Wog Wog

Beetles: Species frequency of extinction was calculated at year 24-25 as follows. First, for each species in each fragment whether a species was originally present was determined and then if it was still present at 25 years. For that species, the number of fragments that transitioned from a presence to an absence between year 5 and year 25 post fragmentation were then summed, and divided by the total number of patches that had that species present at year 5. Extinction frequency, p, was calculated for control patches in the same way. The empirical logit was used to represent the logarithmic odds of extinction: ln((p+0.5)/(1-p+0.5)). Finally, to obtain a change in frequency of extinction in fragments for each species, given the background level of extinction in control patches, the logarithmic odds ratio was calculated by subtracting the logit frequency of extinctions in control patches from the logit frequency of extinction in fragments.

Presence and species richness of understory plants: Herbaceous vegetation was sampled in a 3 x 3 m plot at each site. Each plot consisted of four (75 x 75 cm) quadrats in each corner of the plot; each quadrat was subdivided into 25 (15 x 15 cm) subquadrats. Presence /absence for all flowering plant, ferns, bryophytes and lichens was recorded for each subquadrat. Monitoring was done annually from 1985-1998 and again for the slope plots only in 2010. Species richness per 3 x 3 m plot was calculated for each year. A one-way ANOVA was performed to compare mean 3 x 3 m plot species richness in fragments versus controls in continuous forest for slope plots in each year (n=4 plots per fragment; 72 total plots). For Figure 4 (main text), mean species richness per plot in fragments is expressed as a percentage of mean richness in controls for that year. Filled circles indicate significant differences between controls and fragments for that year.

Tree survival: Repeat tree surveys (1987, 2013) were conducted in each patch, with the sampling strategy adjusted so that approximately the same number of trees were surveyed in each patch type (2418 trees over 12 fragment and 6 continuous forest patches). For each tree, the diameter at 1.4 m above the ground (DBH: diameter at breast height) was recorded. In 1987, trees with DBH greater than 3 cm (i.e. definition of a tree) were permanently labeled and the DBH measured. In 2013, all the trees

labeled in 1987 were relocated and their DBH and mortality were recorded. To divide the data into easily interpretable classes for examining the differential mortality effect on small and large diameter trees, two size diameter classes (3-15 cm and >25 cm) were defined. For Figure 3 (main text), the percentage of trees on fragment edges (0-10 m from edge) that survived in either the small or large diameter class is reported.

Soil nutrients: Soil samples were collected in 1987 prior to the clearing of the matrix area and, therefore, only at 144 pitfall trapping sites within the patches. Soils were collected as bulk samples from the A horizon. Total Organic Carbon was measured using a modified Walkley-Black procedure (Heanes 1984). A second set of soil samples was collected in November, 2012. Soil surface samples were collected at each of 188 pitfall sites (in both patches and matrix). At each site, one of the two insect traps was randomly selected and in 3 locations within 2 m of the trapping site ~25 g of soil was sampled using a metal teaspoon. The three samples were combined into the final bulked-sample. For each of the three samples, soil to a depth of ~4 cm was homogenized in-situ and then the 25 g sample was placed into a plastic zipper-topped bag. Total Organic Carbon and Total Nitrogen were determined by Dumas high temperature combustion on a Leco TruSpec analyzer (Leco 1995). Soil samples were combusted at 950˚C and all gases generated were passed through an infrared detector for carbon and a thermal conductivity cell for nitrogen. All soil results are reported on an oven-dry basis. Figures 3 and 4 (main text) report the percent difference between fragment edges (0 -10 m from edge) and continuous forest fragments for a given year.

<u>Methods for Unpublished Studies – Savannah River Site</u>

Plant species richness: A walk-through survey was conducted annually in June to create a list of all plant species present in each of the 40 experimental fragments; from this list, species richness was calculated for each fragment. Damschen et al. (2006) presents a comparison of connected and unconnected fragments through 2005 and fully describe the sampling methods; Figure 4B (main text) extends this comparison through 2012. For Figure 4B, annual mean richness in connected peripheral fragments is compared to annual mean richness for peripheral unconnected fragments (winged and rectangular). The center fragment in each block was not used in these analyses.

Supplementary Table S1 | Metadata for Figure 3 in the main text. The habitat fragmentation experiment, ecological level of study, and variables of interest for each arrow in Figure 3 are shown below. Comparison describes the data from more fragmented and less fragmented treatments used to calculate effect sizes. Response summarizes the directional impact of the effect. Effect size for each response is calculated as log response ratio (ln(response in more fragmented treatment/response in less fragmented treatment)). The effect sizes are used to create means and ranges in Figure 3. Source describes where the original data are published with full citations in the Supplemental Information References.

Experiment	Ecological Level	Variable	Comparison	Effect Size	Response	Source
BDFFP	Abundance	Birds (insectivorous and frugivorous)	1 ha fragment v. pre-fragmentation	-0.92	Lower abundances in fragment	Stouffer et al. 2006
BDFFP	Species Persistence	Birds	1 ha fragment v. pre-fragmentation	-1.59	Lower persistence (higher extinction rates) in fragments	Stouffer et al. 2011
BDFFP	Species richness	Insectivorous Birds	1 ha fragment v. unfragmented control	-1.06	Lower species richness in fragments	Stratford and Stouffer 1999
BDFFP	Species richness	Butterflies	1 ha fragment v. unfragmented control	-0.39	Lower species richness in fragments	Leidner et al. 2010
BDFFP	Species richness	Dung Beetles	1 ha fragment v. unfragmented control	-0.64	Lower species richness in fragments	Klein 1989
BDFFP	Species richness	Palms	1 ha fragment v. unfragmented control	-0.11	No effect of fragment area on richness	Scariot 1999
BDFFP	Species richness	Myrmecophytic Plants	1 ha fragment v. unfragmented control	-0.07	No effect of fragment area on richness	Bruna et al. 2005
BDFFP	Species richness	Ants on myrmecophytic plants	1 ha fragment v. unfragmented control	-0.59	No effect of fragment area on richness	Bruna et al. 2005
BDFFP	Species richness	Bryophytes	1 ha fragment v. unfragmented control	-0.36	Lower species richness in fragments	Zartman 2003
BDFFP	Community composition	Pioneer tree density (52 species)	Near v. far from edge	-1.16	Higher densities near edge, equates to proportionally lower densities of old growth trees	Laurance et al. 2006b
BDFFP	Community composition	Old growth tree mortality (19 species)	Near v. far from edge	-1.12	Persistence decreased (lower importance) near edge	Laurance et al. 2006a
BDFFP	Nutrient Retention	Plant necromass	Near v. far from edge	0.22	Higher litter and other necromass near edge	Nascimento and Laurance 2004
BDFFP	Productivity	Biomass	Near v. far from edge	-0.10	Lower biomass near edge	Laurance et al. 1997
BDFFP	Trophic Dynamics	Herbivory	1 ha fragment v. unfragmented control	-0.55	Lower herbivory in fragments	Faveri et al. 2008
Kansas	Microclimate	Soil temperature -- winter	Small v. large fragments	0.12	Higher maximum temperatures in small fragments	Unpublished Data, see text
Kansas	Microclimate	Soil temperature -- summer	Small v. large fragments	0.15	Higher maximum temperatures in small fragments	Unpublished Data, see text
Kansas	Residency	Small mammal, deer mice, prop. moved	Small v. large fragments	0.32	Fewer animals move among traps in fragments	Diffendorfer et al. 1995
Kansas	Residency	Small mammal, prairie voles, prop. moved	Small v. large fragments	0.27	Fewer animals move among traps in fragments	Diffendorfer et al. 1995

Experiment	Ecological Level	Variable	Comparison	Effect Size	Response	Source
Kansas	Residency	Small mammal, deer mice, distance moved	Small v. large fragments	-0.73	Animals move farther as fragmentation increased	Diffendorfer et al. 1995
Kansas	Residency	Small mammal, prairie voles, distance moved	Small v. large fragments	-0.77	Animals move farther as fragmentation increased	Diffendorfer et al. 1995
Kansas	Abundance	Spiders	Small v. large fragments	-0.33	Higher densities of spiders in large fragments	Johnson et al. 2010
Kansas	Abundance	Insects	Small v. large fragments	-0.24	Higher insect densities in large fragments	Martinko et al. 2006
Kansas	Abundance	Plants	Small v. large fragments	0.48	Rates of decline toward extinction varied with reproductive mode: e.g. clonal plants decline more quickly in small fragments	Collins et al. 2009
Kansas	Species Richness	Insects	Small v. large fragments	-0.10	Higher richness in large fragments	Martinko et al. 2006
Kansas	Species Richness	Plants	Small v. large fragments	-0.06	Higher local richness (1x1) in large fragments	Cook et al. 2005
Kansas	Community composition	Beta diversity	Small v. large fragments	-0.06	Community similarity lower in fragmented patches	Unpublished data, see Supplementary Methods, consistent with Cook et al. 2005
Kansas	Community composition	Environmental Sorting	Isolated v. connected sample sites	-1.20	Communities reflect environmental gradients more in connected vs. isolated fragments	Alexander et al. 2012
Kansas	Succession Rate	Succession rate (woody plant abundance)	Small v. large fragments	-0.25	Woody plant density higher on large fragments during woody colonization phase 1996-2000	Cook et al. 2005
Kansas	Succession Rate	Succession rate (small mammals)	Small v. large fragments	-0.54	White-footed mice (prefers late-successional habitat) absent in all fragments during early succession; more abundant in large fragments later succession	Schweiger et al. 2000
Kansas	Nutrient Retention	Net N mineralized	Small v. large patches	-0.75	Fragmentation increased net N mineralization	Billings and Gaydess 2008
Kansas	Nutrient Retention	N_2O-N losses	Small v. large patches	-0.94	Fragmentation decreased retention of N_20	Billings and Gaydess 2008
Kansas	Nutrient Retention	Organic Carbon %	Small v. large patches	-0.06	Fragmentation decreased % organic carbon	Billings and Gaydess 2008

Experiment	Ecological Level	Variable	Comparison	Effect Size	Response	Source
Kansas	Trophic Dynamics	Herbivory (oviposition damage by cicadas)	Small v. large patches	-0.31	Trees in large fragments received more slit damage by cicadas	Cook et al. 2001
Wog Wog	Abundance	Beetle abundance and presence (325 species), 5 yrs post fragmentation	0.25 ha, 0.875 ha, and 3.062 ha fragments (edges and cores) v. continuous forest controls	0.23	Declining species were rare, isolated, or predators. Increasing species were: abundant, not isolated, fungivores or detritivores	Davies et al. 2000, 2001, and 2004, see Supplementary Methods
Wog Wog	Species Richness	Understory plant richness, 26 years post fragmentation	Fragments v. continuous forest controls	-0.17	Understory richness declined in fragments	Unpublished data, see Supplementary Methods
Wog Wog	Community composition	Small tree mortality at 26 years	Fragment edges v. fragment cores	0.37	Young trees had increased persistence at fragment edges	Unpublished data, see Supplementary Methods
Wog Wog	Community composition	Large tree mortality at 26 years	Fragment edges v. fragment cores	-0.66	Old trees has reduced persistence at fragment edges	Unpublished data, see Supplementary Methods
Wog Wog	Nutrient Retention	Soil total nitrogen	Small edges v. continuous forest controls	-0.15	Soil nitrogen decreased at edges	Unpublished data, see Supplementary Methods
Wog Wog	Nutrient Retention	Soil total organic carbon	Small edges v. continuous forest controls	-0.18	Soil total organic carbon decreased at edges	Unpublished data, see Supplementary Methods
SRS	Microclimate	Temperature	Edge v. center within fragments	-0.10	Cooler near edge	Johnson and Haddad 2011
SRS	Microclimate	Wind	Connected v. unconnected fragments	-0.29	Lower wind flow among unconnected patches	Damschen et al. 2014
SRS	Movement Between Fragments	Butterflies (2 species), Carpenter bee,	Connected v. unconnected fragments	-1.50	Lower movement between unconnected fragments	Haddad et al. 2011
SRS	Movement Between Fragments	Mammals (2 species)	Connected v. unconnected fragments	-1.17	Lower movement between unconnected fragments	Haddad et al. 2011
SRS	Movement Between Fragments	Bird-dispersed plants (5 species)	Connected v. unconnected fragments	-1.15	Lower movement between unconnected fragments	Haddad et al. 2011
SRS	Movement Between Fragments	Pollen (2 species)	Connected v. unconnected fragments	-0.67	Lower movement between unconnected fragments	Haddad et al. 2011
SRS	Abundance	Butterflies (3 species)	Connected v. unconnected fragments	-0.41	Lower abundances in unconnected fragments	Haddad and Baum 1999
SRS	Abundance	Plants native to longleaf woodlands	Connected v. unconnected fragments	-0.10	Lower abundances in unconnected fragments	Damschen et al. 2006
SRS	Species Richness	Plants	Connected v. unconnected fragments	-0.17	Lower species richness in unconnected fragments	Damschen et al. 2006

Experiment	Ecological Level	Variable	Comparison	Effect Size	Response	Source
SRS	Species Richness	Ground-dwelling arthropods	High-edge v. low-edge fragments	-0.10	Lower species richness in fragments with higher edge	Orrock et al. 2011
SRS	Community Composition	Ground-dwelling arthropods	Connected v. unconnected fragments	0.10	Higher evenness (PIE) in unconnected fragments	Orrock et al. 2011
SRS	Trophic Dynamics	Herbivory	Edge v. center within fragments	-0.32	Lower herbivory near edge	Evans et al. 2012
SRS	Trophic Dynamics	Predation of indigo bunting (bird) eggs/fledglings	Chicks fledged in edgy v. compact fragments	-0.43	Higher predation in edgier fragments	Weldon 2006
SRS	Trophic Dynamics	Predation of seeds	Edge v. center in connected v. unconnected fragments	-0.13	Edge effects stronger in edgier fragments	Orrock and Danielson 2005
SRS	Trophic Dynamics	Predation of seeds (3 species)	Connected v. unconnected fragments	-0.07	Lower seed predation in unconnected fragments	Orrock et al. 2003, Orrock and Damschen 2005
SRS	Pollination	Pollination	Connected v. unconnected fragments	-0.80	Lower fruit set in unconnected fragments	Tewksbury et al. 2002
Moss	Abundance	Microarthropods	Connected v. unconnected fragments	-0.72	Lower in isolated and smaller fragments	Gonzalez et al. 1998, Gonzalez 2000
Moss	Species Persistence	Microarthropod presence/absence	Connected v. unconnected fragments	0.97	Lower (higher extinction) in smaller and isolated fragments	Gilbert et al. 1998, Gonzalez 2000
Moss	Species Richness	Microarthropod species	Connected v. isolated fragments	-0.44	Lower in isolated fragments	Gonzalez 2000
Moss	Nutrient Retention	Total nitrogen	Connected v. Isolated fragments	-1.14	Higher in isolated than connected fragments	Staddon et al. 2010
Moss	Nutrient Retention	Dissolved organic carbon	Connected v. isolated fragments	-0.51	Lower in isolated than connected fragments	Staddon et al. 2010

Supplementary Table S2 | Metadata for Figure 4 in main text. For each panel in Figure 4, the corresponding fragmentation experiment, variables of interest, specific comparison made, and the original source of the data are shown.

Panel	Experiment	Variable	Comparison	Source
Figure 4a	Moss	Arthropod species richness	Fragments v. connected control fragments	Gonzalez 2000
Figure 4a	BDFFP	Bird species richness	1 ha fragments, treatment v. pre-fragmentation	Ferraz et al. 2003
Figure 4a	BDFFP	Butterfly species richness	1 ha fragments, treatment v. pre-fragmentation	Leidner et al. 2010
Figure 4a	Wog Wog	Plant species richness	Fragments v. continuous forest controls, accounting for pre-treatment richness	Unpublished data, see Supplementary Methods
Figure 4b	SRS	Plant species richness	Connected v. unconnected fragments	Damschen et al. 2006; Unpublished data for 2007-present, see Supplementary Methods
Figure 4b	Kansas	Plant species richness	Large v small fragments	Cook et al. 2005
Figure 4c	BDFFP	Plant biomass	Near v. far from edge	Laurance et al. 1997
Figure 4c	Moss	Dissolve carbon	Connected v. isolated fragments	Staddon et al. 2010
Figure 4c	Moss	Total nitrogen	Connected v. isolated fragments	Staddon et al. 2010
Figure 4c	Wog Wog	Total organic carbon	0.25ha fragment edges v. continuous forest controls	Unpublished data, see Supplementary Methods

Supplementary Figure Legends

Supplementary Figure S1 | Map of the Biological Dynamics of Forest Fragmentation Project (BDFFP) experiment and location within Brazil. Experimental fragments and control areas are shown in black. Pasture and regenerating forest is shaded. The remaining white space is largely primary tropical rain forest.

Supplementary Figure S2 | Map of the Kansas Fragmentation experiment. Fragments are outlined with rectangles; plant sampling locations are noted with diamonds.

Supplementary Figure S3 | Map of the Wog Wog experiment and location within Australia.

Supplementary Figure S4 | Map of the Savannah River Site (SRS) experiment showing locations of the eight blocks in the second SRS Corridor experiment within the Savannah River Site, SC, USA. Inset is the design for one experimental block, showing five fragments that are connected or unconnected by a corridor and controls for patch area and shape (rectangular and winged fragments).

Supplementary Figure S5 | Design of the Moss experiment. Landscapes were either continuous (unfragmented) or fragmented. Fragments were connected by corridors or unconnected (with or without ‘broken’ control corridors).

Supplementary Figure S6 | Design of the Metatron experiment with 48 enclosed fragments and adjoining enclosed corridors. Environmental conditions are experimentally controlled through an automated system.

Supplementary Figure S7 | Map of the SAFE experiment and location within Borneo (after Ewers et al. 2011). Experimental fragments and corridors (riparian strips) are located within landscapes of varying human land use.

Supplementary Figure S1

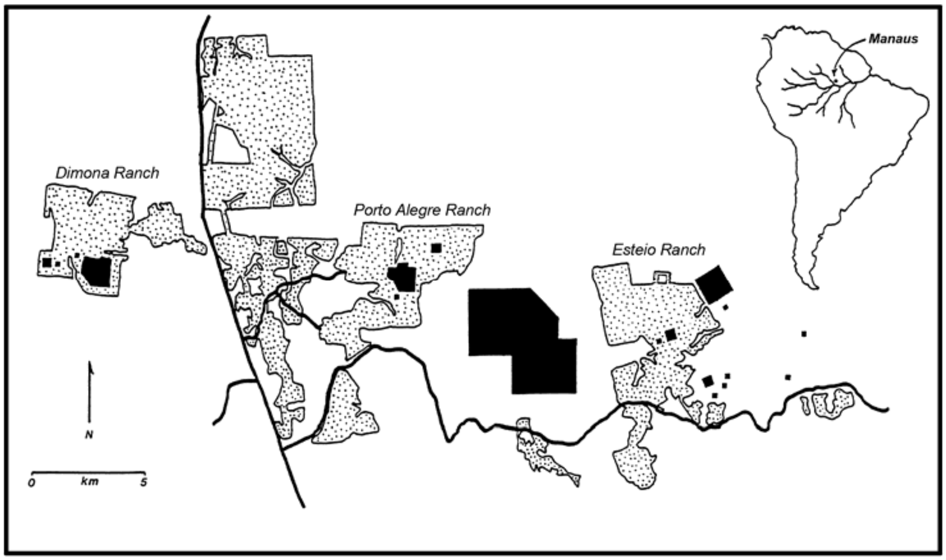

Supplementary Figure S1

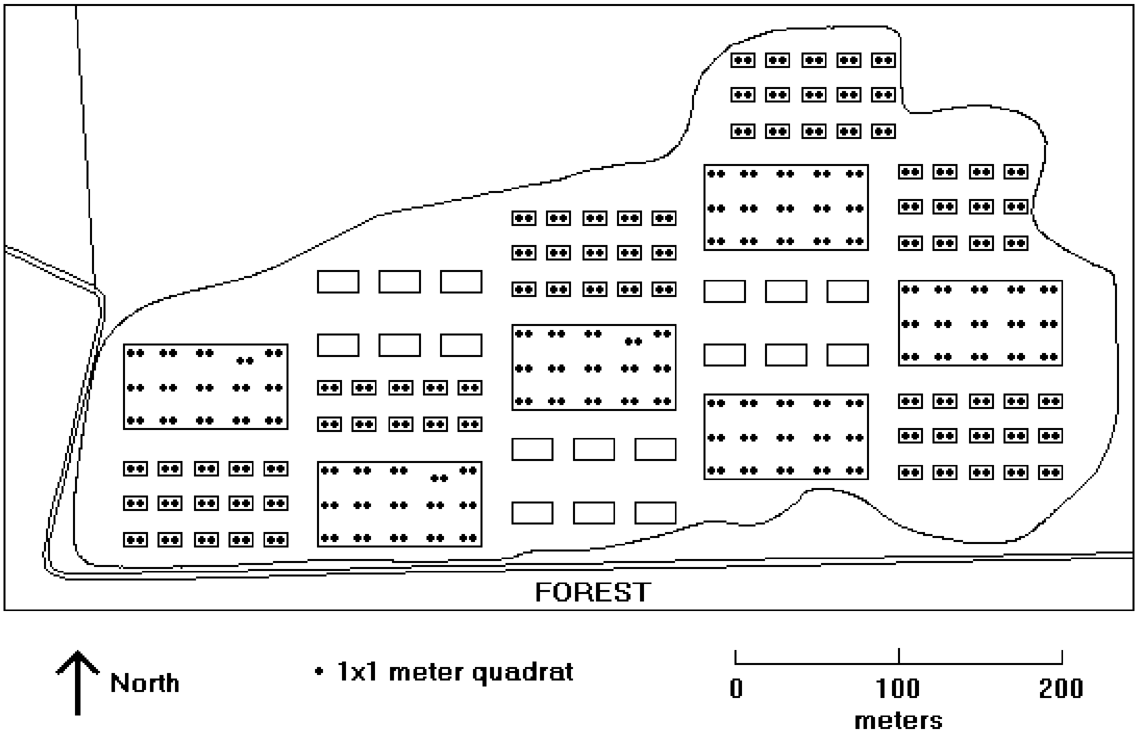

Supplementary Figure S3

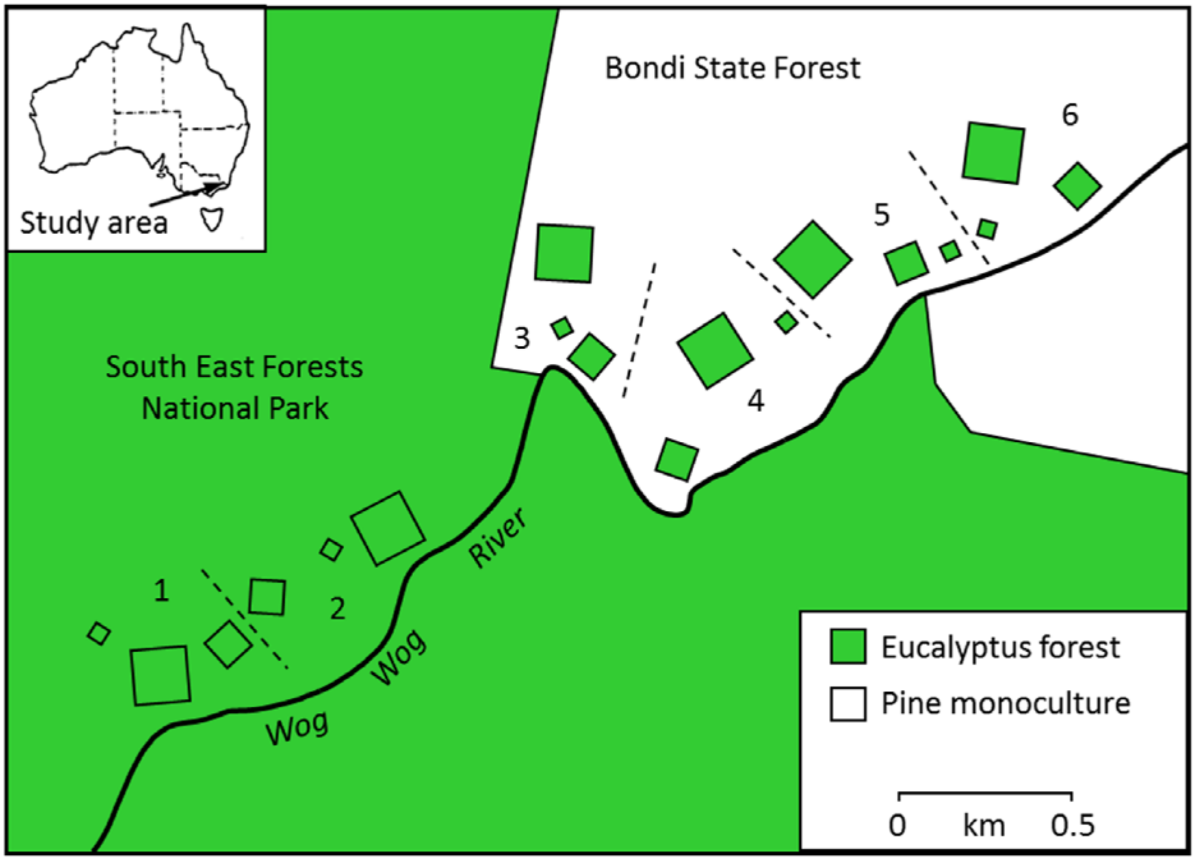

Supplementary Figure S4

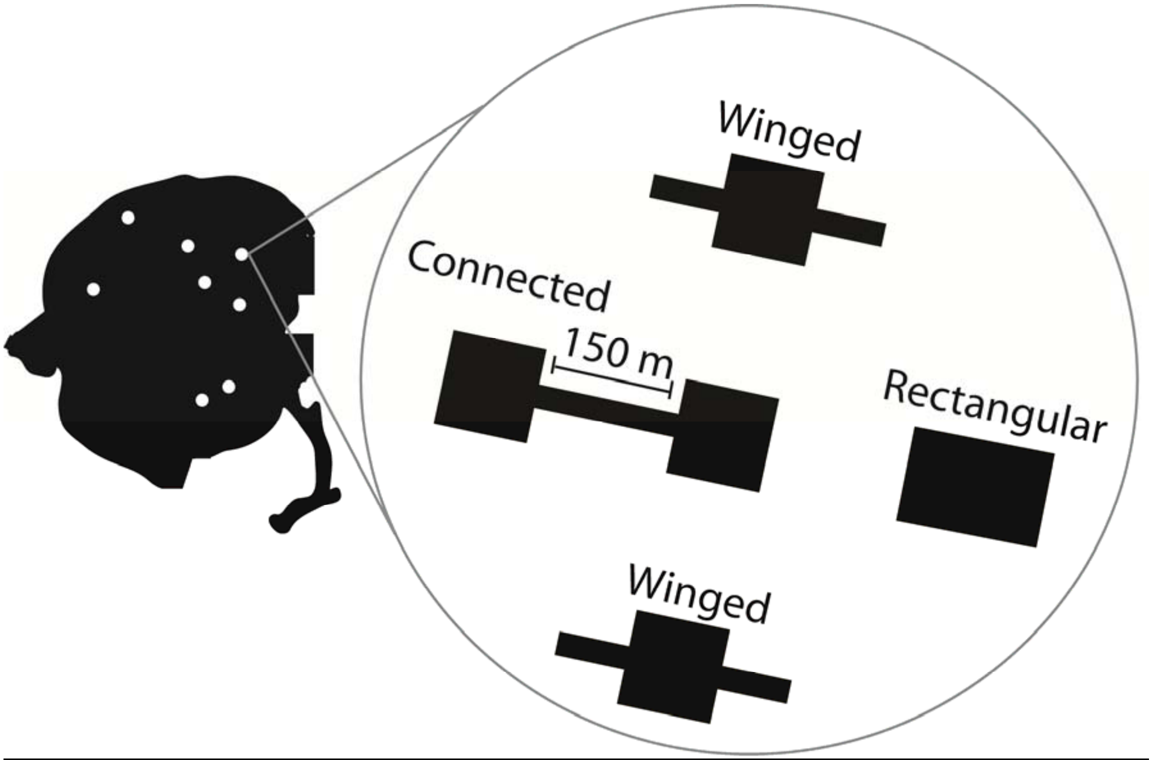

Supplementary Figure S5

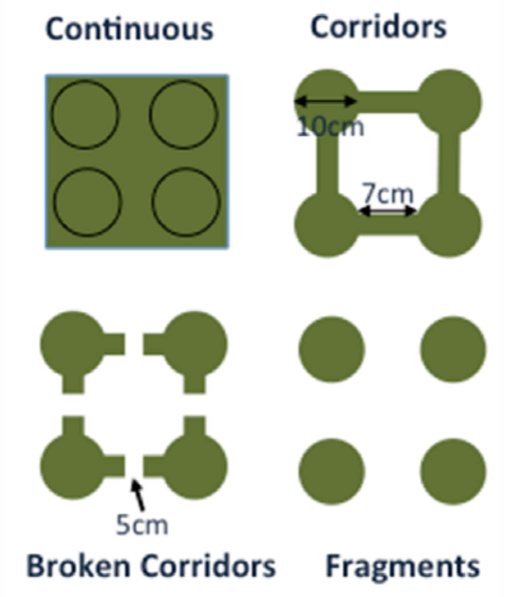

Supplementary Figure S6

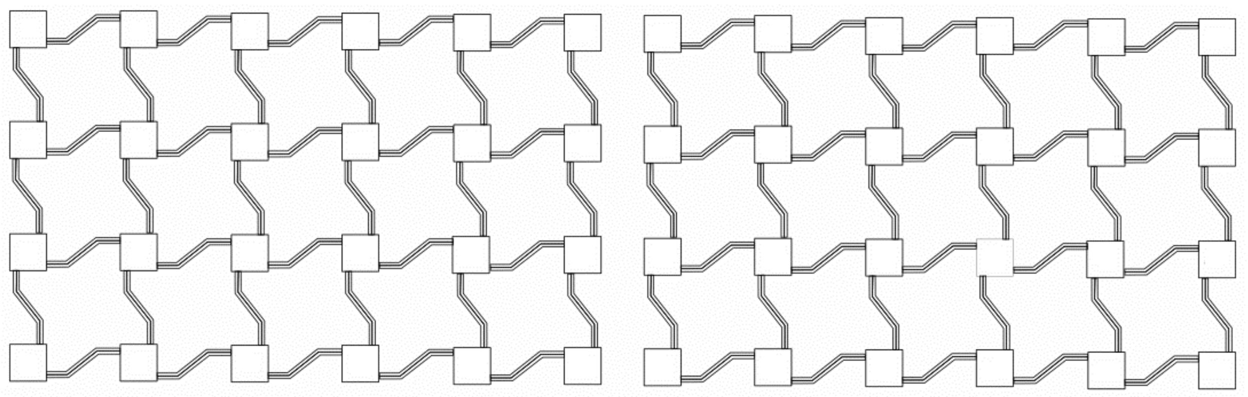

Supplementary Figure S7

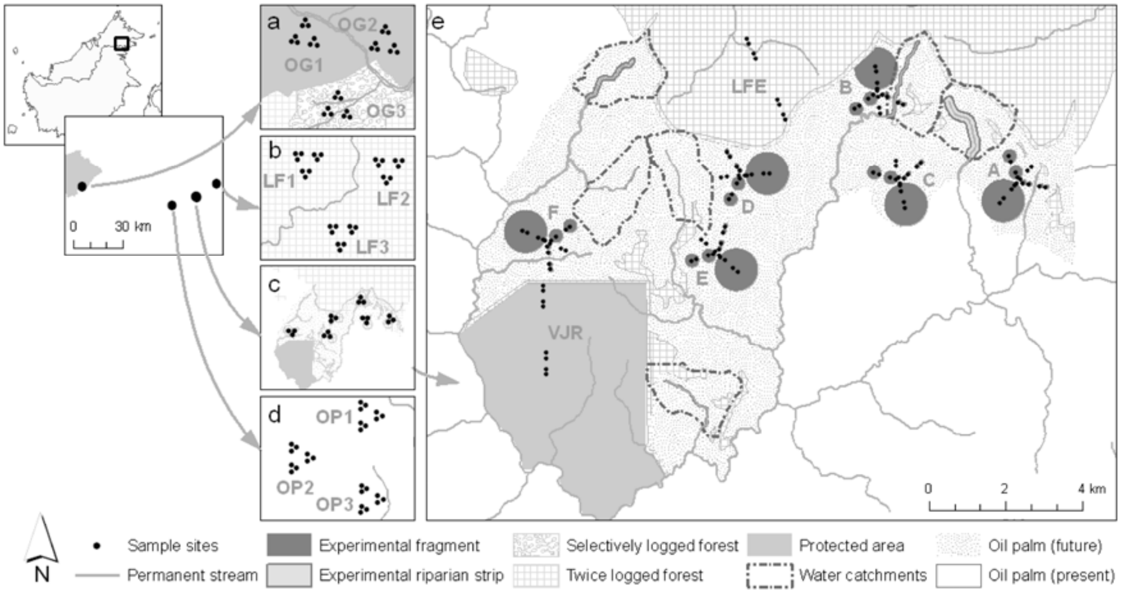

Supplementary References

Instituto Nacional de Pesquisas Espaciais (2009) PRODES: Assessment of deforestation in Brazilian Amazonia. Available at

SOS Mata Atlântica, Instituto Nacional de Pesquisas Espaciais (2008) Atlas dos remanescentes florestais da Mata Atlântica, período de 2000 a 2005. .

Alexander, H. M., B. L. Foster, F. Ballantyne, C. D. Collins, J. Antonovics, and R. D. Holt. 2012. Metapopulations and metacommunities: combining spatial and temporal perspectives in plant ecology. Journal of Ecology **100**:88-103.

Anderson, M. J., K. E. Ellingsen, and B. H. McArdle. 2006. Multivariate dispersion as a measure of beta diversity. Ecology Letters **9**:683-693.

Austin, M. P. and A. O. Nicholls. 1988. Species associations within herbaceous vegetation in an Australian eucalypt forest. Pages 95-114 *in* H. J. During, M. J. A. Werger, and J. H. Willems, editors. Diversity and pattern in plant communities. SPB Academic Publishing, The Hague, The Netherlands.

Belward, A. S. 1996. The IGBP-DIS Global 1 Km Land Cover Data Set “DISCover”: Proposal and Implementation Plans: Report of the Land Recover Working Group of IGBP-DIS. IGBP-DIS.

Billings, S. A. and E. A. Gaydess. 2008. Soil nitrogen and carbon dynamics in a fragmented landscape experiencing forest succession. Landscape Ecology **23**:581-593.

Brudvig, L. A., S. A. Wagner, and E. I. Damschen. 2012. Corridors promote fire via connectivity and edge effects. Ecological Applications **22**:937-946.

Bruna, E. M., H. L. Vasconcelos, and S. Heredia. 2005. The effect of habitat fragmentation on communities of mutualists: Amazonian ants and their host plants. Biological Conservation **124**:209-216.

Câmara, G., D. D. M. Valeriano, and J. V. Soares. 2006. Metodologia para o Cálculo da Taxa Anual de Desmatamento na Amazônia Legal (PRODES) [Methodology for the Calculation of Annual Deforestation rates in the Legal Amazon (PRODES)] Instituto Nacional de Pesquisas Espaciais, São José dos Campos, Brazil.

Clarke, K. R. and R. N. Gorley. 2006. Primer v6 Permanova+.

Collins, C. D., R. D. Holt, and B. L. Foster. 2009. Patch size effects on plant species decline in an experimentally fragmented landscape. Ecology **90**:2577-2588.

Cook, W. M., R. D. Holt, and J. Yao. 2001. Spatial variability in oviposition damage by periodical cicadas in a fragmented landscape. Oecologia **127**:51-61.

Cook, W. M., J. Yao, B. L. Foster, R. D. Holt, and L. B. Patrick. 2005. Secondary succession in an experimentally fragmented landscape: Community patterns across space and time. Ecology **86**:1267-1279.

Damschen, E. I., D. V. Baker, G. Bohrer, R. Nathan, J. L. Orrock, J. R. Turner, L. A. Brudvig, D. J. Levey, N. M. Haddad, and J. J. Tewksbury. 2014. Habitat fragmentation and corridors affect wind dynamics and seed dispersal in open habitats. Proceedings of the National Academy of Sciences **111**:3484-3489.

Damschen, E. I., N. M. Haddad, J. L. Orrock, J. J. Tewksbury, and D. J. Levey. 2006. Corridors increase plant species richness at large scales. Science **313**:1284-1286.

Davies, K. F. 1999. The responses of beetles to experimental forest fragmentation. Australian National University.

Davies, K. F. and C. R. Margules. 1998. Effects of habitat fragmentation on carabid beetles: experimental evidence. Journal of Animal Ecology **67**:460-471.

Davies, K. F., C. R. Margules, and J. F. Lawrence. 2004. A synergistic effect puts rare, specialized species at greater risk of extinction. Ecology **85**:265-271.

Davies, K. F., C. R. Margules, and K. F. Lawrence. 2000. Which traits of species predict population declines in experimental forest fragments? Ecology **81**:1450-1461.

Davies, K. F., B. A. Melbourne, and C. R. Margules. 2001. Effects of within- and between-patch processes on community dynamics in a fragmentation experiment. Ecology **82**:1830-1846.

Diffendorfer, J. E., M. S. Gaines, and R. D. Holt. 1995. Habitat Fragmentation and Movements of 3 Small Mammals (Sigmodon, Microtus, and Peromyscus). Ecology **76**:827-839.

DiMiceli, C., M. Carroll, R. Sohlberg, C. Huang, M. Hansen, and J. Townshend. 2011. Annual Global Automated MODIS Vegetation Continuous Fields (MOD44B) at 250 m Spatial Resolution for Data

Years Beginning Day 65, 2000–2010, Collection 5 Percent Tree Cover. University of Maryland, College Park.
Evans, D. M., N. E. Turley, D. J. Levey, and J. J. Tewksbury. 2012. Habitat patch shape, not corridors, determines herbivory and fruit production of an annual plant. Ecology **93**:1016-1025.
Ewers, R. M., R. K. Didham, L. Fahrig, G. Ferraz, A. Hector, R. D. Holt, V. Kapos, G. Reynolds, W. Sinun, J. L. Snaddon, and E. C. Turner. 2011. A large-scale forest fragmentation experiment: the Stability of Altered Forest Ecosystems Project. Philosophical Transactions of the Royal Society B-Biological Sciences **366**:3292-3302.
Faveri, S. B., H. L. Vasconcelos, and R. Dirzo. 2008. Effects of Amazonian forest fragmentation on the interaction between plants, insect herbivores, and their natural enemies. Journal of Tropical Ecology **24**:57-64.
Gilbert, F., A. Gonzalez, and I. Evans-Freke. 1998. Corridors maintain species richness in the fragmented landscapes of a microecosystem. Proceedings of the Royal Society of London Series B-Biological Sciences **265**:577-582.
Gonzalez, A. 2000. Community relaxation in fragmented landscapes: the relation between species richness, area and age. Ecology Letters **3**:441-448.
Gonzalez, A., J. H. Lawton, F. S. Gilbert, T. M. Blackburn, and I. Evans-Freke. 1998. Metapopulation dynamics, abundance, and distribution in a microecosystem. Science **281**:2045-2047.
Haddad, N. M. 1999. Corridor and distance effects on interpatch movements: a landscape experiment with butterflies. Ecological Applications **9**:612-622.
Haddad, N. M., B. Hudgens, E.I. Damschen, D.J. Levey, J.L. Orrock, J. J. Tewksbury, and A. J. Weldon. 2011. Assessing positive and negative ecological effects of corridors.*in* J. Liu, V. Hull, A. T. Morzillo, and J. A. Wiens, editors. Sources, sinks, and sustainability. Cambridge University Press, Cambridge, UK.
Haddad, N. M. and K. A. Baum. 1999. An experimental test of corridor effects on butterfly densities. Ecological Applications **9**:623-633.
Heanes, D. L. 1984. Determination of total organic C in soils by an improved chromi acid digestion and spectrophotometric procedure. Communications in Soil Science and Plant Analysis **15**:1191-1213.
Holt, R. D., G. R. Robinson, and M. S. Gaines. 1995. Vegetation dynamics in an experimentally fragmented landscape. Ecology **76**:1610-1624.
Johnson, B. L. and N. M. Haddad. 2011. Edge effects, not connectivity, determine the incidence and development of a foliar fungal plant disease. Ecology **92**:1551-1558.
Johnson, J. B., R. H. Hagen, and E. A. Martinko. 2010. Effect of Succession and Habitat Area on Wandering Spider (Araneae) Abundance in an Experimental Landscape. Journal of the Kansas Entomological Society **83**:141-153.
Klein, B. C. 1989. Effects of forest fragmentation on dung and carrion beetle communities in central Amazonia. Ecology **70**:1715-1725.
Laurance, W. F., J. L. C. Camargo, R. C. C. Luizao, S. G. Laurance, S. L. Pimm, E. M. Bruna, P. C. Stouffer, G. B. Williamson, J. Benitez-Malvido, H. L. Vasconcelos, K. S. Van Houtan, C. E. Zartman, S. A. Boyle, R. K. Didham, A. Andrade, and T. E. Lovejoy. 2011. The fate of Amazonian forest fragments: A 32-year investigation. Biological Conservation **144**:56-67.
Laurance, W. F., S. G. Laurance, L. V. Ferreira, J. M. RankindeMerona, C. Gascon, and T. E. Lovejoy. 1997. Biomass collapse in Amazonian forest fragments. Science **278**:1117-1118.
Laurance, W. F., H. E. M. Nascimento, S. G. Laurance, A. Andrade, J. Ribeiro, J. P. Giraldo, T. E. Lovejoy, R. Condit, J. Chave, K. E. Harms, and S. D'Angelo. 2006a. Rapid decay of tree-community composition in Amazonian forest fragments. Proceedings of the National Academy of Sciences of the United States of America **103**:19010-19014.
Laurance, W. F., H. E. M. Nascimento, S. G. Laurance, A. C. Andrade, P. M. Fearnside, J. E. L. Ribeiro, and R. L. Capretz. 2006b. Rain forest fragmentation and the proliferation of successional trees. Ecology **87**:469-482.
Leco. 1995. Instruction Manual FP-2000 Protein/Nitrogen Analyses. Instrumentation for characterisation of organic/inorganic materials and microstructural analysis.
Legrand, D., O. Guillaume, M. Baguette, J. Cote, A. Trochet, O. Calvez, S. Zajitschek, F. Zajitschek, J. Lecomte, Q. Benard, J. Le Galliard, and J. Clobert. 2012. The Metatron: an experimental system to study dispersal and metaecosystems for terrestrial organisms. Nature Methods **9**:828-833.

Lehner, B., K. Verdin, and A. Jarvis. 2008. New global hydrography derived from spaceborne elevation data. EOS Trans. Am. Geophys. Union **89**:93-94.
Leidner, A. K., N. M. Haddad, and T. E. Lovejoy. 2010. Does Tropical Forest Fragmentation Increase Long-Term Variability of Butterfly Communities? PLoS One **5**.
Lindo, Z., J. Whiteley, and A. Gonzalez. 2012. Traits explain community disassembly and trophic contraction following experimental environmental change. Global Change Biology **18**:2448-2457.
Margules, C. R. 1992. The Wog-Wog habitat fragmentation experiment. Environmental Conservation **19**:316-325.
Martinko, E. A., R. H. Hagen, and J. A. Griffith. 2006. Successional change in the insect community of a fragmented landscape. Landscape Ecology **21**:711-721.
Nascimento, H. E. M. and W. F. Laurance. 2004. Biomass dynamics in Amazonian forest fragments. Ecological Applications **14**:S127-S138.
Olson, D. M., E. Dinerstein, E. D. Wikramanayake, N. D. Burgess, G. V. N. Powell, E. C. Underwood, J. A. D'Amico, I. Itoua, H. E. Strand, J. C. Morrison, C. J. Loucks, T. F. Allnutt, T. H. Ricketts, Y. Kura, J. F. Lamoreux, W. W. Wettengel, P. Hedao, and K. R. Kassem. 2001. Terrestrial ecoregions of the worlds: A new map of life on Earth. Bioscience **51**:933-938.
Orrock, J. L., G. R. Curler, B. J. Danielson, and D. R. Coyle. 2011. Large-scale experimental landscapes reveal distinctive effects of patch shape and connectivity on arthropod communities. Landscape Ecology **26**:1361-1372.
Orrock, J. L. and E. I. Damschen. 2005. Corridors cause differential seed predation. Ecological Applications **15**:793-798.
Orrock, J. L. and B. J. Danielson. 2005. Patch shape, connectivity, and foraging by oldfield mice (Peromyscus polionotus). Journal of Mammalogy **86**:569-575.
Orrock, J. L., B. J. Danielson, M. J. Burns, and D. J. Levey. 2003. Spatial ecology of predator-prey interactions: corridors and patch shape influence seed predation. Ecology **84**:2589-2599.
Ribeiro, M. C., J. P. Metzger, A. C. Martensen, F. J. Ponzoni, and M. M. Hirota. 2009. The Brazilian Atlantic Forest: How much is left, and how is the remaining forest distributed? Implications for conservation. Biological Conservation **142**:1141-1153.
Scariot, A. 1999. Forest fragmentation effects on palm diversity in central Amazonia. Journal of Ecology **87**:66-76.
Schweiger, E. W., J. E. Diffendorfer, R. D. Holt, R. Pierotti, and M. S. Gaines. 2000. The interaction of habitat fragmentation plant, and small mammal succession in an old field. Ecological Monographs **70**:383-400.
Sexton, J. O., X. P. Song, M. Feng, P. Noojipady, A. Anand, C. Q. Huang, D. H. Kim, K. M. Collins, S. Channan, C. DiMiceli, and J. R. Townshend. 2013. Global, 30-m resolution continuous fields of tree cover: Landsat-based rescaling of MODIS vegetation continuous fields with lidar-based estimates of error. International Journal of Digital Earth **6**:427-448.
Staddon, P., Z. Lindo, P. D. Crittenden, F. Gilbert, and A. Gonzalez. 2010. Connectivity, non-random extinction and ecosystem function in experimental metacommunities. Ecology Letters **13**:543-552.
Stouffer, P. C., R. O. Bierregaard, C. Strong, and T. E. Lovejoy. 2006. Long-term landscape change and bird abundance in Amazonian rainforest fragments. Conservation Biology **20**:1212-1223.
Stouffer, P. C., E. I. Johnson, R. O. Bierregaard, and T. E. Lovejoy. 2011. Understory Bird Communities in Amazonian Rainforest Fragments: Species Turnover through 25 Years Post-Isolation in Recovering Landscapes. PLoS One **6**.
Stratford, J. A. and P. C. Stouffer. 1999. Local extinctions of terrestrial insectivorous birds in a fragmented landscape near Manaus, Brazil. Conservation Biology **13**:1416-1423.
Tewksbury, J. J., D. J. Levey, N. M. Haddad, S. Sargent, J. L. Orrock, A. Weldon, B. J. Danielson, J. Brinkerhoff, E. I. Damschen, and P. Townsend. 2002. Corridors affect plants, animals, and their interactions in fragmented landscapes. Proceedings of the National Academy of Sciences USA **99**:12923-12926.
Trochet, A., D. Legrand, N. Larranaga, S. Ducatez, O. Calvez, J. Cote, J. Clobert, and M. Baguette. 2013. Population sex ratio and dispersal in experimental, two-patch metapopulations of butterflies. Journal of Animal Ecology **82**:946-955.
Weldon, A. J. 2006. How corridors reduce Indigo Bunting nest success. Conservation Biology **20**:1300-1305.

Zartman, C. E. 2003. Habitat fragmentation impacts on epiphyllous bryophyte communities in central Amazonia. Ecology **84**:948-954.
